# Study on the cognitive effects of eye movement desensitization and reprocessing: proposed active mechanisms

**DOI:** 10.3389/fncom.2026.1751744

**Published:** 2026-08-10

**Authors:** Tammy Soliman, Matthew Heller, Samuel Girguis

**Affiliations:** Quantitative and Qualitative Behavioral Lab, Research Psychology and Data Analysis Program, Department of Psychology, College of Education and Behavioral Sciences, Azusa Pacific University, Azusa, CA, United States

**Keywords:** post-traumatic stress disorder, EMDR, default mode network, Interleukin-6, Interleukin-10, Neuropeptide Y, Artificial Neural Network (ANN), Quantum-AI

## Abstract

**Introduction:**

Post-traumatic stress disorder (PTSD) is characterized by intrusive memories and an impaired resilience framework, which often leads to chronic psychological distress. Eye Movement Desensitization and Reprocessing (EMDR) is an emerging therapeutic approach targeting PTSD, yet the precise neurobiological mechanisms remain inadequately defined. Recent studies suggest immune modulation, mainly through molecules such as Interleukin-6 (IL-6), Interleukin-10 (IL-10), Neuropeptide Y (NPY), and Oxytocin (OXY), as well as additional proteins, may play a key role in longitudinal outcomes. This study investigated the roles of immune factors, systemic interactions, and neurobiological functions, including the Default Mode Network (DMN) and B-cell regulation, in PTSD remission and EMDR efficacy.

**Methodology:**

A systematic dry-lab analytical approach was employed using Artificial Neural Network (ANN) modeling and dynamic pathway analysis to reveal neurobiological factors, immune function, and neural network differences involved in PTSD remission and EMDR functionality. Publicly available gene expression datasets were analyzed, focusing on key pathways, interactions, and gene loci related to immune regulation, neurobiological modulation, and inflammatory response. Key molecules, including IL-6, IL-10, NPY, and OXY, were examined for potential contributions to neurobiological resilience, therapeutic response, and immune function in response to adverse stimuli.

**Results:**

The ANN analysis revealed that immunological function, notably through IL-10 and B-cell activity, plays a prominent role in PTSD remission, EMDR outcomes, and resilience. IL-10 emerged as central to B-cell differentiation and proliferation regarding trauma and resilience, indicating its potential as a biomarker for PTSD within the first year of symptom onset. Additional analyses implicated IL-6 and NPY as critical to longitudinal neurobiological resilience mechanisms. Interestingly, while OXY was initially classified as significant for social bonding and PTSD remission, analysis showed this gene only played a secondary mediating role, aligning with recent findings that OXY is not strictly necessary for prosocial outcomes such as PTSD remission.

**Conclusion:**

This study suggests that IL-10, IL-6, and NPY are key neuroimmune modulators in PTSD remission and may explain EMDR functionality. Immunologic activity may also explain variations in EMDR outcomes, such as therapeutic success, resistance, and relapse rates. These findings underscore the potential of targeted co-occurring immunotherapies, possible objective PTSD tests, potential objective measures for EMDR outcomes, and personalized approaches for enhanced treatment.

## 1 Introduction

Memory formation depends on coordinated cortical, hippocampal, and endocrine processes, yet their interactions with neuronal, immune, and molecular mechanisms remain not completely understood. Unimodal sensory cortices process environmental stimuli and route information via specific brain structures to specific brain regions. Representations are integrated to form complete memories (Stickgold, 2002; Liang et al., 2013; Kitamura et al., 2017; Williams et al., 2023; Wang et al., 2024). Baddeley’s working memory (WM) model summarizes the interactions among these elements (Baddeley and Hitch, 1974).

**FIGURE 1:**
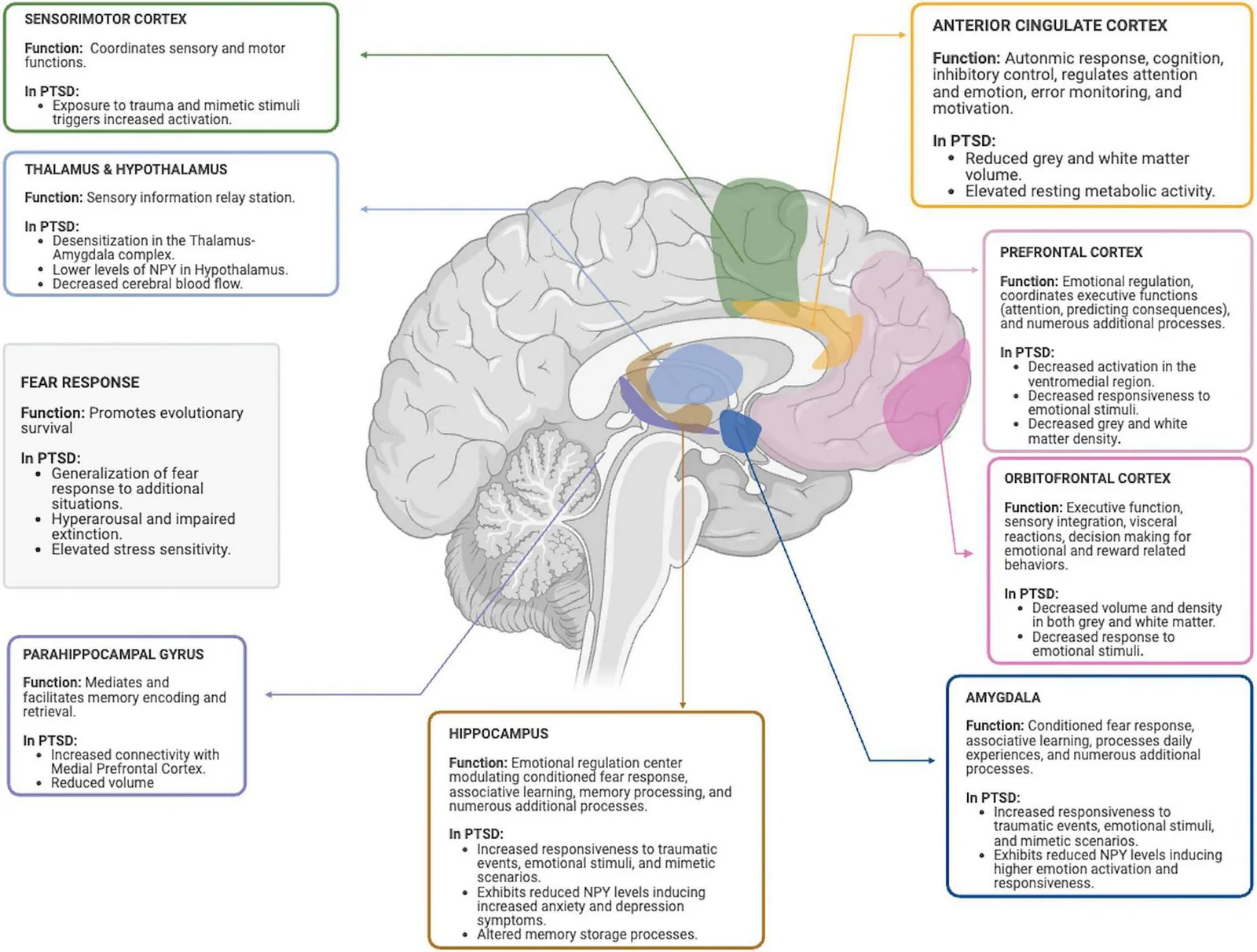
Brain schema of the limbic system involvement in PTSD. The prefrontal cortex and hippocampus, both densely connected to the amygdala, play critical roles in conditioned fear and emotional learning. The prefrontal cortex is involved in reactivating emotional associations and shows reduced responsiveness and density in PTSD. The hippocampus, which mediates responses to contextual cues and explicit memories of trauma, exhibits reduced volume and responsiveness in PTSD. These changes may increase amygdala activation, resulting in heightened stress sensitivity, generalized fear, and impaired extinction. Other regions, including the anterior cingulate cortex, orbitofrontal cortex, parahippocampal gyrus, thalamus, and sensorimotor cortex, also contribute to fear regulation and PTSD. Created with BioRender (https://BioRender.com). NPY, Neuropeptide Y.

Traumatic events can disrupt cognitive processing and impair memory encoding, resulting in distressing memories. In post-traumatic stress disorder (PTSD), disruptions in the amygdala and hypothalamic-pituitary-adrenal (HPA) axis, along with additional brain regions, alter memory processing, with neurotrophic molecular factors such as Acetyl-CoA synthetase 2 (ACSS2) and Pituitary adenylate cyclase-activating polypeptide (PACAP) influencing recall and trauma encoding, respectively, often leading to enhanced arousal for episodic memories and altered semantic memory dynamics (Stickgold, 2002; Liang et al., 2013; Mews et al., 2017; Marshall et al., 2020; Alexander et al., 2022; Lawrence and Scofield, 2024). Such disruptions may result in PTSD, where traumatic memories are continually reexperienced via neuronal excitation and activation (Figure 1).

Emerging behavioral neuroscience supports the finding that multiple memory mechanisms mediate maladaptive memory creation, expression, and remission (Tabibnia, 2020). Studies implicate Interleukin-6 (IL-6), Interleukin-10 (IL-10), Neuropeptide Y (NPY), the HPA axis, and Z-DNA morphology, among other neurotrophic molecular factors. Epigenetic modifications in binding domains may occur following trauma exposure, influencing the formation of adverse memories, SNS neuronal clusters, and systemic function. The implications of these factors in PTSD onset and remission suggest differential activation patterns and memory mechanisms that influence individual susceptibility and therapeutic response (Parrish et al., 2010; Bowers et al., 2012; Wu et al., 2013; Gibbs et al., 2014; Bountress et al., 2018; Furukawa et al., 2020; Vinkers et al., 2021; Alexander et al., 2022).

Given this multifactorial neurobiological framework, therapeutic approaches such as Eye Movement Desensitization and Reprocessing (EMDR) aim to modulate these disrupted systems and restore adaptive memory processing. However, the precise mechanism is not completely understood.

Eye Movement Desensitization and Reprocessing is an effective intervention for PTSD that engages key neural circuits and memory systems, including the amygdala, hippocampus, and HPA-axis networks, to promote iterative deactivation of trauma-related responses. Despite its clinical efficacy, the underlying mechanisms remain unclear, as emerging evidence suggests that neuroimmune, molecular, and epigenetic factors–such as NPY, ACSS2, CDK5, and immune signaling–may all influence treatment response, relapse, and long-term outcomes (Stickgold, 2002; Landin-Romero et al., 2013; Li et al., 2017; Shapiro, 2018; Brenna et al., 2019; Adams et al., 2020; Hwang et al., 2025).

The predominant hypothesis underlying EMDR is the Adaptive Information Processing (AIP) model. The model asserts that bilateral stimulation (BS) removes processing barriers, enabling real-time reorganization, rerouting, and storage of memory (Hase et al., 2017). However, this model lacks an explanation of specific mechanisms that impact PTSD remission and relapse (Shapiro, 2018). Additional variations of the AIP and extinction training models have yet to fully integrate neurobiological components implicated in both PTSD pathology and EMDR facilitated remission (Maxfield et al., 2008; Adams et al., 2018; de Voogd et al., 2018; Laure Wadji et al., 2022; Carvalho Silva et al., 2024).

Recent genome-wide association studies indicate that polygenic interactions, along with epigenetic and immune-related mechanisms, contribute to PTSD susceptibility, onset, and remission (Nievergelt et al., 2015, 2019; Olff, 2017; Vinkers et al., 2021). These findings support multivariate frameworks, including the tripartite model, which integrates neurobiological, epigenetic, and allostatic processes in both PTSD development and recovery (Tabibnia, 2020). Despite advances in knowledge, the precise immune-related mechanisms underlying PTSD and memory function in EMDR response remain insufficiently defined across populations.

Evidence further suggests that EMDR efficacy depends on stimulus type, exposure duration, and level of neurobiological engagement. Trauma-congruent stimuli enhances symptom remission more effectively than non-matching inputs, supporting the roles of unimodal sensory pathways and the Default Mode Network (DMN) in memory processing (Van Den Hout et al., 2011; Matthijssen et al., 2017). Emerging computational and neuroscience models, including AIP, WM, and tripartite frameworks, highlight neural plasticity, methylation, epigenetic modulation, immune regulation, and allostatic load activation as key mechanisms underpinning EMDR (Akiki et al., 2018; Tabibnia, 2020).

Similar to anesthesia, EMDR remains mechanistically unresolved, with unclear optimal stimulation parameters and limited integration of immune function and multiomics data. High relapse rates (30%–40%) and variability in treatment response further underscore the need for comprehensive systemic models that incorporate neuroimmune dynamics between innate and adaptive systems, genetic predisposition, and systemic network interactions such as “Z-DNA morphology” and “death fold” protein configurations to explain therapeutic outcomes, which may better inform precision interventions (Jonas et al., 2013; Difede et al., 2014; Peterson et al., 2017).

This study aimed to determine whether extrinsic neuroimmune systems influence PTSD remission, therapeutic resistance, and EMDR outcomes. It was hypothesized that potential B-cell–mediated immune responses and interactions contribute to bilateral stimulation efficacy and PTSD pathology, thereby clarifying underlying neurobiological mechanisms. To test this, we applied hierarchical clustering, network integration algorithms, Artificial Neural Network (ANN) analysis, and Monte Carlo simulations to evaluate key signaling molecules, including IL-6, IL-10, NPY, ACSS2, and CDK5.

## 2 Materials and methods

### 2.1 Identification and classification of study cohorts

Extensive functional association data were compiled from a comprehensive literature search across PubMed, Google Scholar, NIH, WHO, VAERS, and PRR databases, using combinations of “PTSD,” “remission,” “onset,” “symptoms,” “recovery,” and “gene” as key search terms. A functional enrichment analysis using Gene Ontology (GO) was performed to enhance model predictive power and identify additional target genes. Additionally, supplemental data from the Mouse Genome Informatics^1^ were incorporated to assess convergence between homologous sample profiles and pathways. An international online database comprised of laboratory mice and other animals was incorporated into the analysis. Queries were filtered along “Homo Sapiens” to include integrated genetic, genomic, and biological data, facilitating a multimodal study of human health and disease. All included studies are listed in Supplementary Materials 2–5, and the extracted gene datasets are summarized in Supplementary Material 1.

Target genetic information was extracted and compiled into a review to conduct a hierarchical cluster analysis (Feder et al., 2009; Niitsu et al., 2019; Maul et al., 2020; Kuan et al., 2021). In this study, *n* denotes the number of genes within each dataset or gene set, and a total of target genes were analyzed.

Clustering and dimensionality reduction methods (K-means, hierarchical clustering, factor analysis, and PCA) identified two primary cohorts: PTSD-associated genes (*n* = 66) and B-cell-associated genes (*n* = 174). These were further subdivided into PTSD remission (*n* = 34) and onset (*n* = 32), and into B-cell up-regulation (*n* = 107) and down-regulation (*n* = 67).

**FIGURE 2:**
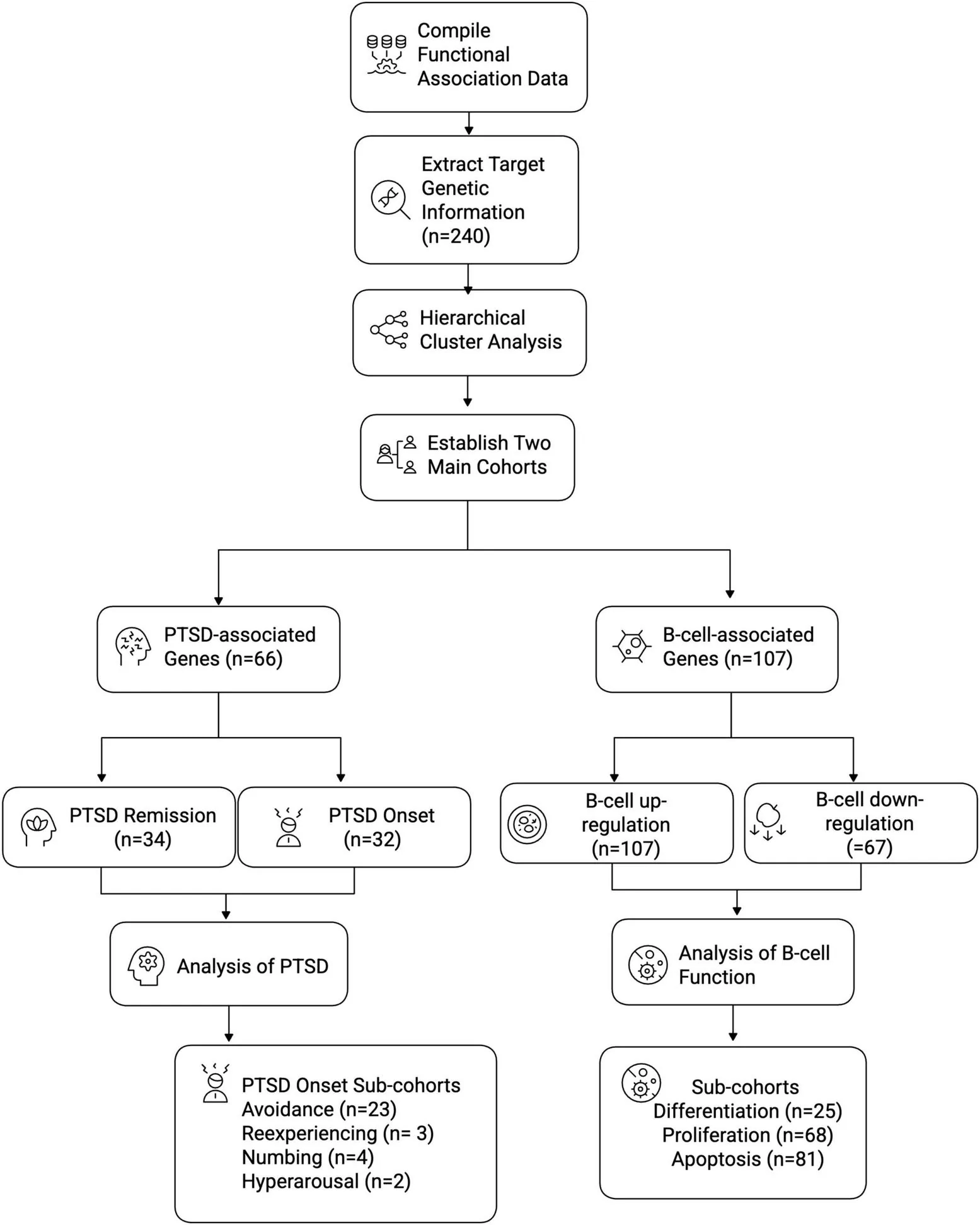
Study cohort identification and classification. Functional association data from peer-reviewed literature, genome-wide association studies, and curated gene datasets were integrated to compile a set of 240 genes for hierarchical clustering. Two primary cohorts were defined: PTSD-associated genes (*n* = 66) and B-cell-associated genes (*n* = 174), using K-means clustering, hierarchical clustering, factor analysis, and principal component analysis (PCA). Subgroup stratification identified PTSD remission (*n* = 34) and onset (*n* = 32), as well as B-cell up-regulation (*n* = 107) and down-regulation (*n* = 67). Secondary analyses further resolved PTSD domains and B-cell functional subpopulations; however, collinearity led to consolidation of PTSD domains, and apoptosis-related B-cell pathways were excluded from subgroup analysis while retaining multifunctional genes. Functional enrichment using Gene Ontology and integration of Mouse Genome Informatics data enhanced pathway characterization and supported multimodal analysis of gene interactions and biological function. Created with Napkin AI (https://www.napkin.ai/).

Further analysis of PTSD classes resulted in the sub-cohorts of remission, avoidance, reexperiencing, numbing, and hyperarousal. In contrast, B-cell function resulted in sub-cohorts of up-regulation differentiation, down-regulation differentiation, up-regulation proliferation, down-regulation proliferation, up-regulation apoptosis, down-regulation, and apoptosis (Figure 2 and Supplementary Material 1, respectively). After completing factor analysis and PCA, five PTSD cohorts (Remission, Avoidance, Re-experiencing, Numbing, and Hyperarousal) remained for systematic analysis to determine the nature of interdependencies, connections, interactions, and functionality.

During secondary analysis using K-Means clustering and factor analysis, the PTSD Onset subgroup revealed four domains that exhibited redundancy and co-expression among genes; these were therefore retained in the sample as a single cohort for dimensionality reduction. The B-cell-associated genes cohorts revealed additional subpopulations, which were independent for PTSD Remission and PTSD Onset. Among B-cell-associated gene sub-cohorts, apoptosis was excluded due to the extensive processing requirements. However, individual genes with broader functional roles were retained in the PTSD Remission cohort (Mustafa et al., 2024).

### 2.2 Data sources, cohort interactions, and pathway connections

Initial gene input groups, cohort clusters consisting of PTSD onset, PTSD remission, and B-cell-associated genes cohorts, were analyzed using a nested multiple association network integration algorithm and an ANN (de Oliveira et al., 2018; Keaton et al., 2021; Renner et al., 2022; Toft et al., 2022; Speakman et al., 2023).

Output groups comprising intermediary nodes, centrality, connection type, connection density, and pathways were generated, then systematically parsed, sorted, compared, and partitioned for downstream analysis of cascading effects.

Pathway-level vectors and comparative analyses were applied to explore potential associations, interactions, and functional relationships between gene sets. The observed effects support the potential clinical relevance of the findings within this exploratory framework for both clinical and non-clinical populations; however, results should be interpreted as credibly inferential, given the exploratory design and the gene-level nature of the analyses.

### 2.3 Secondary analysis

A multiple association network integration algorithm and ANN analysis were performed to explore potential connections between gene loci, neurobiological correlates, and immune function.

The PTSD Remission group (*n* = 34) was analyzed and compared against B-cell up-regulation differentiation (*n* = 20), B-cell down-regulation differentiation (*n* = 5), B-cell up-regulation proliferation (*n* = 47), and B-cell down-regulation proliferation (*n* = 21) groups to explore potential connections between post-traumatic growth and immune function.

Systemic gene function was explored using GeneMANIA^®2^, an interactive software that supports dynamic testing across multiple gene functional types, predictive analyses, and prioritization for functional assays (Mostafavi et al., 2008; Franz et al., 2018). From this analysis, connections between the input and output groups emerged. Proposed intermediary groups demonstrated direct connections and critical pathways, centrality for specific genes and receptors, and mechanisms of action.

A four-dimensional NxM matrix was used to analyze input groups comprising PTSD remission–associated genes and output vectors derived from B-cell gene cohorts, including up- and down-regulated genes involved in differentiation and proliferation. Both forward propagation and backpropagation was used to confirm analytical findings for all systemic analyses. This approach enabled the identification of intermediary cohorts, revealing additional pathways, supplementary genes, associated receptors, and potential binding domains.

Each analysis assessed the strength of connections within and between pathways and gene interactions by assigning specific colors and line densities. This intermediary cohort linked PTSD remission to B-cell functionality and revealed a cohort of potential systemic mechanisms of action.

### 2.4 Third analysis

A final analysis was conducted to assess the inferential importance of IL-6, IL-10, NPY, OXY, associated receptors, binding domains, and additional genes included in the preceding cohorts.

This follow-up analysis aimed to determine whether convergence among the significantly identified markers was present. The first array was defined as “PTSD-Associated Remission Genes,” while the second was termed “B-cell Associated Genes.”

This final stage explored gene set permutations, combinations, and network dynamics using Monte Carlo simulation, which repeatedly sampled values at random to assess centrality and convergence, while a chaotic noise parameter simulated environmental dynamics and longitudinal trends.

## 3 Results

**FIGURE 3:**
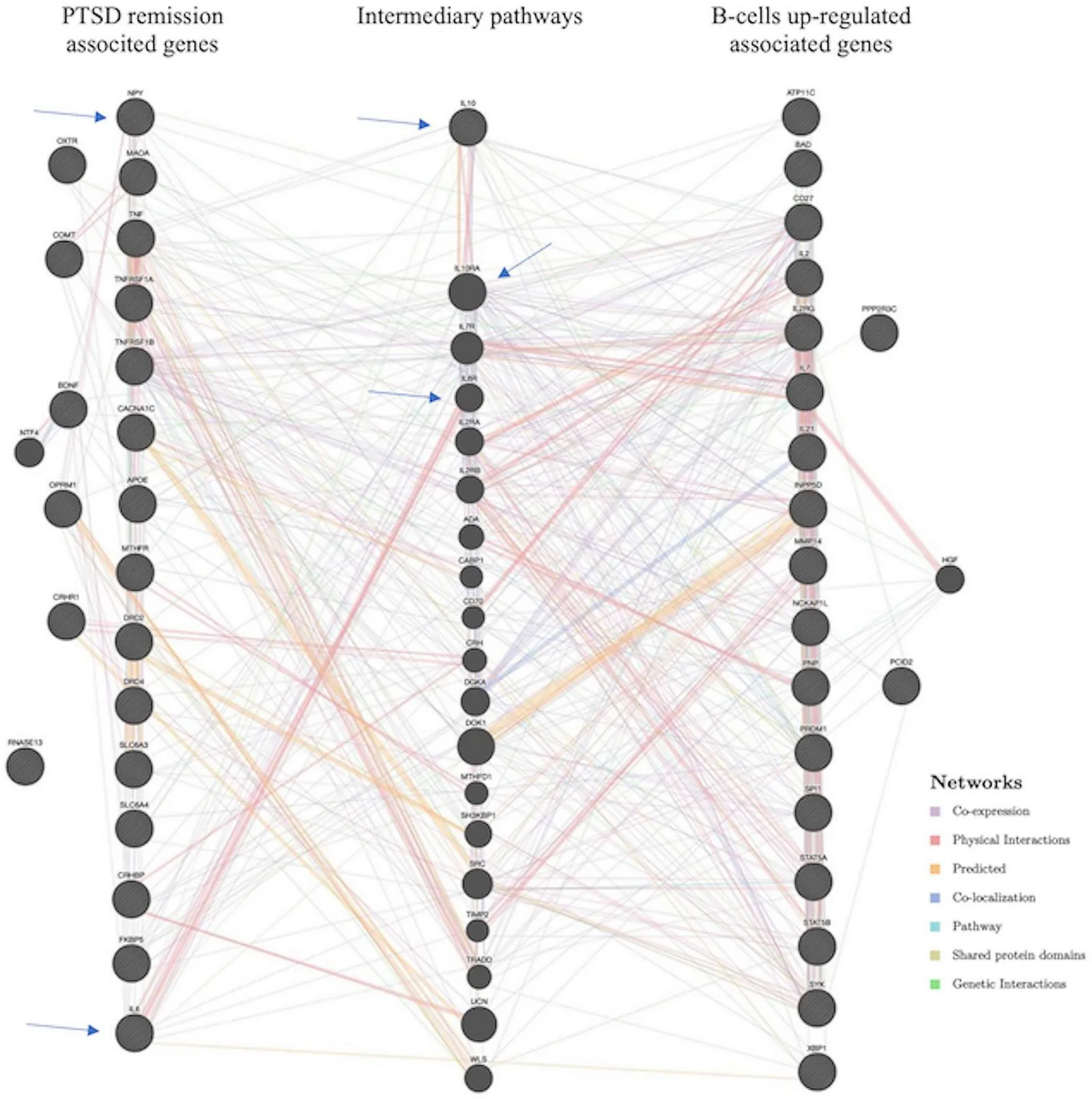
Post-traumatic stress disorder (PTSD) remission via B-cell up-regulation differentiation. The figure illustrates network interactions between PTSD remission-associated genes and B-cell differentiation pathways, as identified using GeneMANIA^®^ software. Intermediary pathways establish mechanistic links between immune modulation and PTSD remission. Colored lines represent different types of gene interactions: co-expression (purple), genetic interactions (green), pathways (blue), physical interactions (red), shared protein domains (orange), and predicted interactions (yellow). Blue arrows highlight key regulatory genes and vector pathways, indicating strong associations between PTSD remission and B-cell differentiation during up-regulation. The layout is supported by GeneMANIA^®^ (Supplementary Material 2).

**FIGURE 4:**
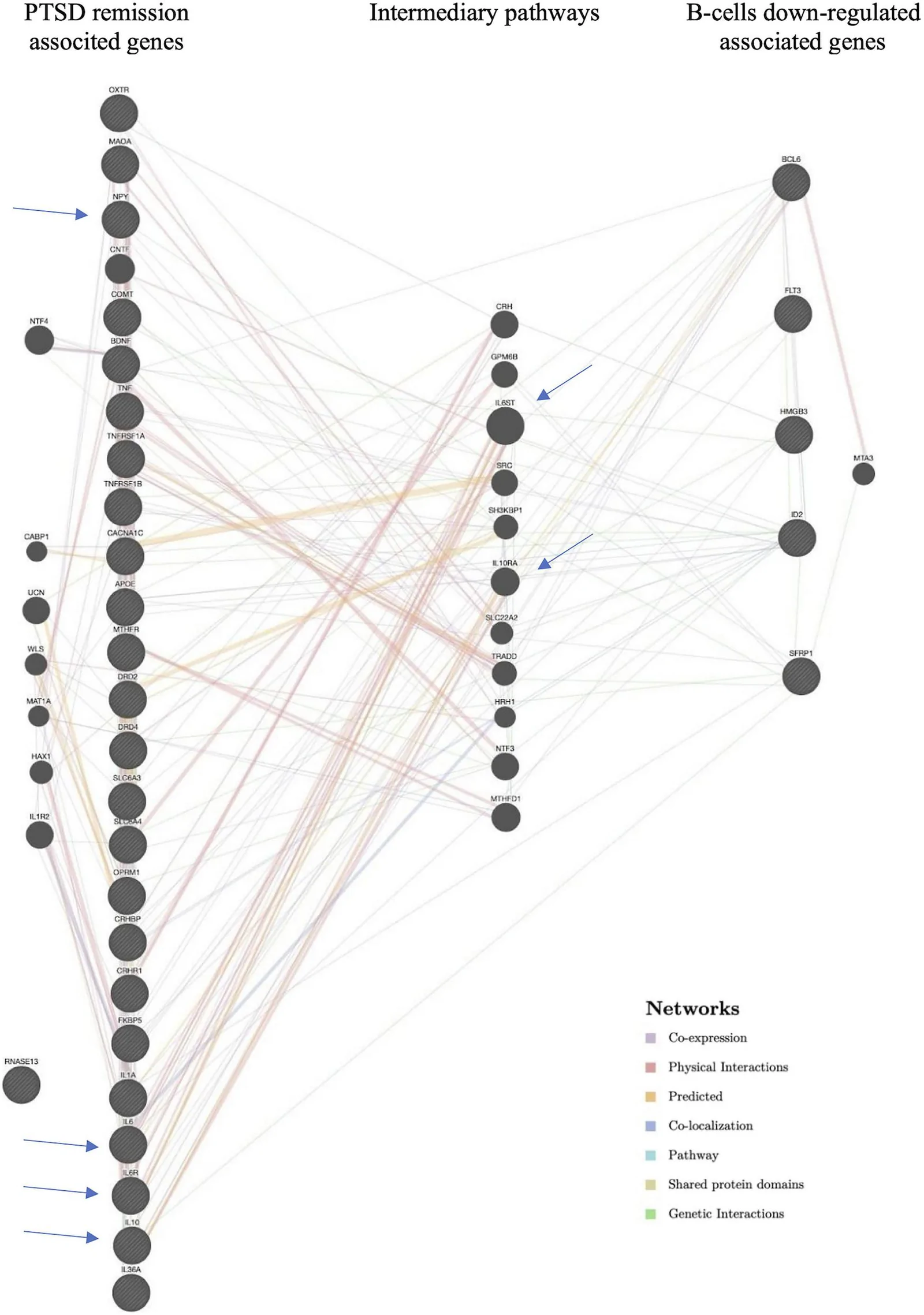
Post-traumatic stress disorder (PTSD) remission via B-cell down-regulation differentiation. The figure illustrates network interactions between PTSD remission-associated genes and B-cell differentiation pathways during downregulation, as determined using GeneMANIA^®^ software. The analysis reveals intermediary pathways that may influence immune function in PTSD remission. Colored lines represent different types of gene interactions: co-expression (purple), genetic interactions (green), pathways (blue), physical interactions (red), shared protein domains (orange), and predicted interactions (yellow). Blue arrows highlight key regulatory genes and vector pathways, indicating strong associations between PTSD remission and B-cell differentiation during down-regulation. The layout is supported by GeneMANIA^®^ (Supplementary Material 3).

**FIGURE 5:**
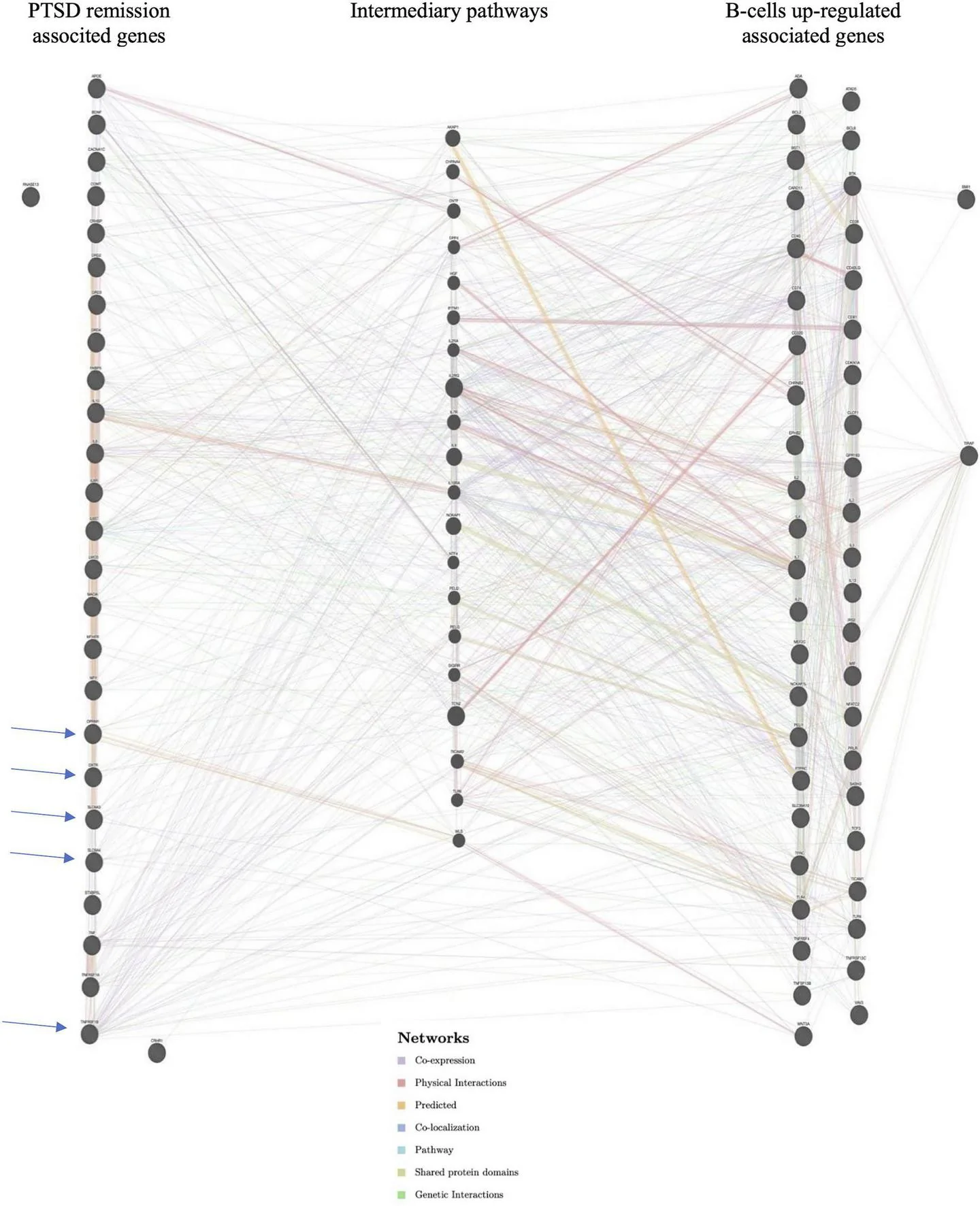
Post-traumatic stress disorder (PTSD) remission via B-cell up-regulation proliferation. The figure illustrates the network interactions between PTSD remission-associated genes and B-cell proliferation pathways, as identified using GeneMANIA^®^ software. The diagram highlights intermediary pathways involved in immune response and neurobiological function in PTSD remission. Colored lines represent different types of gene interactions: co-expression (purple), genetic interactions (green), pathways (blue), physical interactions (red), shared protein domains (orange), and predicted interactions (yellow). Blue arrows highlight key regulatory genes and vector pathways, indicating strong associations between PTSD remission and B-cell proliferation during up-regulation. The layout is supported by GeneMANIA^®^ (Supplementary Material 4).

**FIGURE 6:**
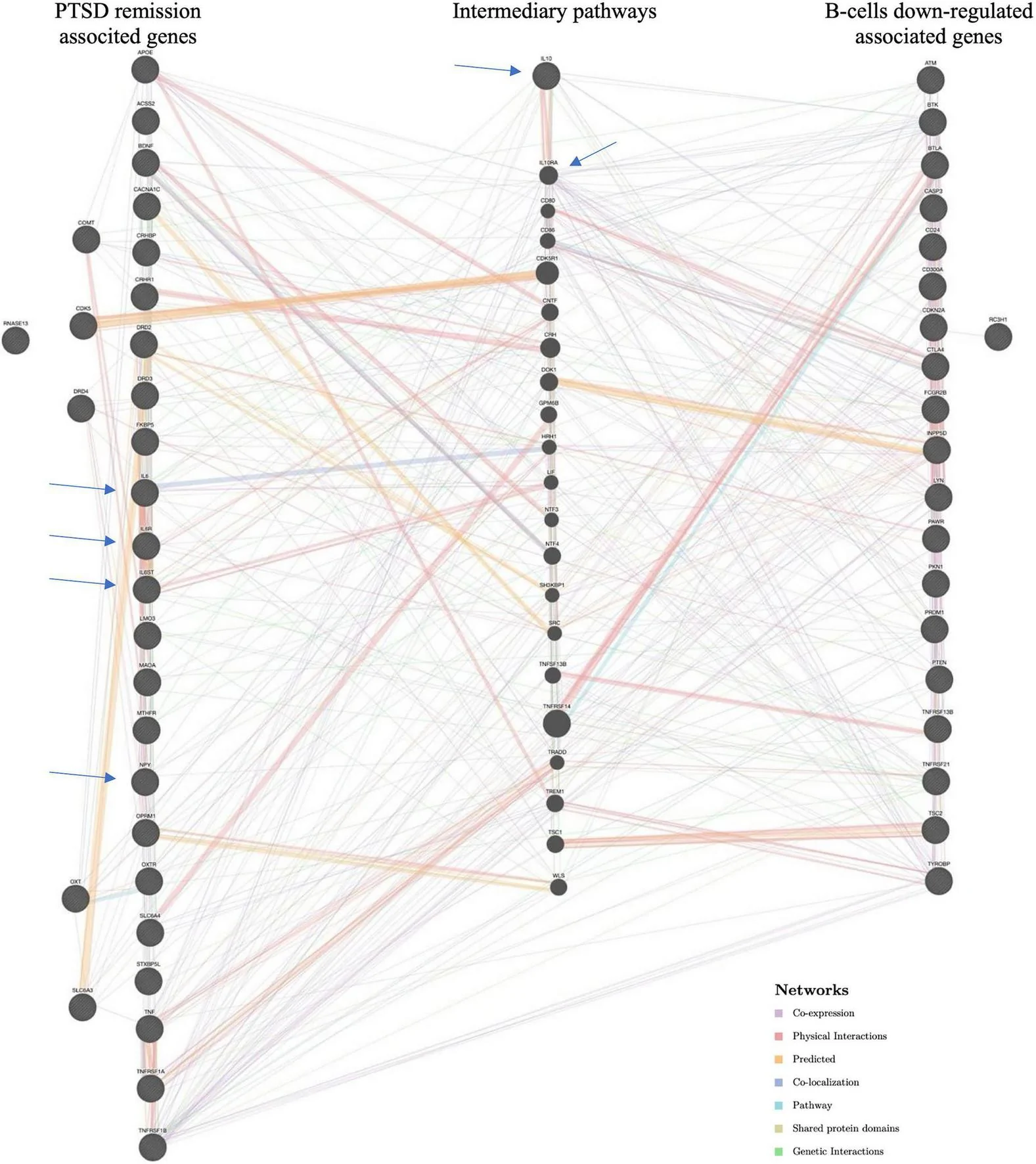
Post-traumatic stress disorder (PTSD) remission via B-cell down-regulation proliferation. The figure illustrates the network interactions between PTSD remission-associated genes and B-cell proliferation pathways during downregulation, as determined using GeneMANIA^®^ software. The diagram highlights intermediary pathways involved in immune response and neurobiological function in PTSD remission. Colored lines represent different types of gene interactions: co-expression (purple), genetic interactions (green), pathways (blue), physical interactions (red), shared protein domains (orange), and predicted interactions (yellow). Blue arrows highlight key regulatory genes and vector pathways, indicating strong associations between PTSD remission and B-cell proliferation during down-regulation. The layout is supported by GeneMANIA^®^ (Supplementary Material 5).

Results from the four GeneMANIA^®^ analyses identified multiple pathways between PTSD remission and B-cell-associated genes (Figures 3–6). Several indirect and inferential mechanisms also emerged, supporting the functional relationship between PTSD remission and B-cell regulation systems. B-cell up- and down-regulation in differentiation and proliferation emerged as central factors in PTSD remission in relation to IL-10 (Figures 3, 6, respectively).

While the results suggest potential clinical relevance, they are based on a gene-level analysis and should therefore be interpreted as credibly inferential, with mild caution.

### 3.1 IL-10 as a critical factor in PTSD remission and B-cell function

Interleukin-10 demonstrated strong convergence, significance, superposition, and centrality within the network linking PTSD remission and B-cell function, supporting its role as a key associative mediator between these systems. This nodal cluster appeared within a direct pathway connecting PTSD remission to B-cell up-regulation and differentiation (Figure 6, blue arrow) and was present in both PTSD remission–associated and B-cell–associated gene sets (Figure 4, blue arrow), with IL-10RA identified as a relevant receptor component. Although no direct IL-10 interactions were observed in Figure 5, IL-10RA was positioned within an intermediate pathway linking PTSD remission to B-cell down-regulation during proliferation (Figure 6, blue arrow), suggesting coordinated network involvement.

Pathway and vector analyses indicated that IL-10 up-regulation was associated with B-cell differentiation in PTSD remission (Figure 3), whereas IL-10 downregulation was linked to B-cell proliferation (Figure 6, blue arrows). These patterns highlight a bidirectional association, consistent with the regulatory role of IL-10 in balancing inflammatory and anti-inflammatory processes during early and iterative PTSD stages (2 weeks, 1 year, respectively).

### 3.2 IL-6 and its role in PTSD remission and immune function

Interleukin-6 showed significant associations with PTSD remission through both direct (Figure 3, blue arrow) and indirect network associations. IL-6R emerged as a significant intermediary component connecting PTSD remission with B-cell up-regulation differentiation. IL-6 was also present within PTSD remission-associated genes (Figure 4, blue arrow), with IL-6ST contributing as an additional pathway component.

Although IL-6 and associated receptors showed only mild centrality and convergence across all networks, their co-occurrence in key pathways suggests a coordinated role in the inflammatory response and immune-related processes. In particular, IL-6, IL-6R, and IL-6ST were all associated with PTSD remission within the proliferation-related network (Figure 6), supporting their involvement in neuroimmune regulation. As expected, direct or causal IL-6 interactions were not significantly identified (Figure 5).

### 3.3 Neuropeptide Y as a resilience factor in PTSD

Neuropeptide Y emerged as a key resilience factor, showing direct and indirect association with PTSD remission across analyses (Figures 3–6). This 36-amino-acid polypeptide demonstrated direct interactions with IL-2 and co-expression with IL-2RG, supporting involvement from neuroimmune signaling.

Neuropeptide Y (Figures 3, 4, blue arrow) directly links to PTSD remission and connects to B-cell up-regulation differentiation through a direct pathway (green line). In Figure 6, NPY (blue arrow) connects to PTSD remission-associated genes that were co-expressed with B-cell down-regulation and proliferation networks, reinforcing its role within coordinated immune responses. However, a direct NPY interaction was not identified through this analysis (Figure 5).

### 3.4 Oxytocin: a limited role in PTSD remission

This analysis indicated that OXY was not consistently, significantly, or convergently associated with direct effects across pathways. OXTR demonstrated only indirect associations for PTSD remission (Figure 3) via secondary interactions with primary receptors and proteins. OXY appeared as a supportive, indirect, and inferential PTSD remission-associated gene (Figure 4, blue arrow), with moderating links to B-Cell differentiation networks.

Direct OXY interactions were not identified through this analysis (Figure 5). However, Figure 6 (blue arrow) showed secondary associations between OXY and PTSD remission, with B-cell down-regulation and proliferation as potential supportive mediation mechanisms.

## 4 Discussion

### 4.1 Role of Interleukin groups in B-cell regulation and PTSD remission

This study provided a detailed, multilevel analysis of the relationships among B-cell function, immune modulation, and PTSD remission, identifying IL-10 and IL-6 as potential mediators of immune adaptation for trauma remission. This analysis showed that IL-10 emerged as a critical component of PTSD remission, influencing both B-cell differentiation and proliferation, consistent with its established role as a key anti-inflammatory cytokine (Stanic et al., 2015; Kim et al., 2020; Toft et al., 2022). IL-10 plays a dual role by modulating B-cell activation, promoting B-cell up-regulation during proliferation while simultaneously down-regulating B-cell differentiation pathways (Fluckiger et al., 1993). This dynamic regulatory function suggests that IL-10 regulates the equilibrium between immune activation, suppression, and adaptation, a process that is potentially pivotal for long-term screening, recovery, and resilience for PTSD (Kim et al., 2020).

Similarly, IL-6 was identified as a potential key secondary immunomodulator in PTSD remission, exhibiting an association with IL-10 and linking with additional TNF signaling pathways. IL-6 is widely recognized for its role in inflammatory response, yet its presence in B-cell differentiation pathways suggests it may also contribute to immune modification in PTSD and EMDR (Neylan and O’Donovan, 2019). Notably, IL-6 up-regulation was associated with B-cell differentiation in PTSD remission, implicating this signaling protein in immune system modification following trauma exposure (Katrinli et al., 2022). While IL-10 predominantly exerted an anti-inflammatory effect, IL-6 may serve as a transitional or translational factor between innate and adaptive immunity. IL-6 potentially facilitates immune cell memory responses that later evolve into regulatory phases crucial for PTSD remission and long-term immune system memory (de Oliveira et al., 2018; Govindula et al., 2023).

These dynamic analyses support the potential for interactions among IL-10, IL-6, and secondary target genes, as well as between B-cell systems, further contributing to the growing understanding that PTSD remission involves neurological reorganization and methylation, immune memory, and memory cell activation (de Oliveira et al., 2018). The role of IL-10RA as a significant intermediary receptor pathway supports the hypothesis that epigenetic modifications in immune signaling may actively influence PTSD outcomes as well (Golubeva et al., 2024). Both Interleukin groups may act as cooccurring regulatory mechanisms that mediate between innate and adaptive immunity to promote resilience. These findings suggest that therapeutic strategies targeting IL-10 and IL-6 pathways, such as Dopaminergic and Histaminergic neurons, could enhance PTSD treatment and EMDR efficacy, particularly in interventions engaging bilateral stimulation which may indirectly modulate immune function. This finding also potentially explains why the role of PACAP in onset, remission, and demographic dimorphism was found to be significant.

### 4.2 Neuropeptide Y as a resilience factor in PTSD

This analysis showed that NPY is directly linked to PTSD remission via both gene interactions and co-expression, which facilitates stress adaptation, particularly through its association with B-cell up-regulation differentiation, contributing to immune resilience by promoting adaptive immune responses following trauma exposure (Bowers et al., 2012). A systematic analysis incorporating GENEMANIA^®^ showed that NPY is also associated with B-cell down-regulation differentiation genes. This dual role in up- and down-regulation pathways reinforces its role as a stabilizing factor for the immune system. This NPY association aligns with existing research suggesting that it plays a protective role in stress resilience by modulating HPA axis activity and neuroimmune communication (Reichmann and Holzer, 2016). This may be due to protein orientation, superposition, morphology, folding, or binding domain.

Additionally, NPY was observed in co-expression networks with B-cell down-regulation proliferation genes, further supporting its involvement in immune recalibration during PTSD remission. The absence of direct interaction suggests that NPY’s influence may be more prominent in differentiation pathways rather than proliferation mechanisms, potentially acting through an indirect regulatory network.

These findings reinforce the hypothesis that NPY serves as a bridge between stress response systems and immune adaptation during recovery. This potential role in modulating B-cell differentiation and proliferation suggests an additional potential therapeutic target for enhancing longitudinal resilience in PTSD treatment. Given the well-established effects of NPY on reducing anxiety, regulating autonomic function, and promoting synaptic plasticity, NPY may contribute to the long-term stabilization of PTSD remission, particularly in response to interventions such as EMDR that engage neuroimmune pathways.

### 4.3 The limited role of oxytocin in PTSD remission

Interestingly, results indicated that OXY did not play a direct and critical role in the observed pathways for PTSD remission. This outcome contrasts with prior inferential findings that posited OXY and associated receptors as essential for prosocial behaviors during PTSD recovery (Sack et al., 2017; Flanagan and Mitchell, 2019; Carmassi et al., 2021). However, these results align with more recent studies demonstrating that OXY was not strictly necessary for prosocial outcomes in both mammalian and animal models (Berendzen et al., 2023).

Gene-edited mouse models lacking an OXY receptor exhibited levels of prosocial behavior, such as successful pair bonding and child-rearing, similar to those of wild-type groups. These results are consistent with human studies indicating that exogenous OXY administration does not consistently and significantly affect memory formation or trauma sequelae (Sack et al., 2017; Flanagan and Mitchell, 2019; Carmassi et al., 2021). These findings challenge the longstanding belief that OXY-mediated social bonding is a critical mechanism in PTSD remission and suggest that EMDR’s efficacy may not depend exclusively on OXY-related pathways. Instead, the therapeutic relationship between clinician and patient, often proposed as a critical mechanism underpinning EMDR efficacy, may rely on alternative neurobiological processes unrelated to OXY, dyadic clinician-patient hierarchies, or secondarily related alternate social bonding structures (Witteveen et al., 2019; Le Dorze et al., 2020).

### 4.4 B-cell activation in PTSD: contrasting findings and implications

These analyses draw further connections between B-cell activation and PTSD outcomes, with IL-10 emerging as a prominent factor potentially influencing both processes and outcomes. This result aligns with research findings reporting elevated B-cell activation in gene-edited traumatized mouse models, suggesting an immune response component associated with adversity (Lynall et al., 2021). However, these findings also raise questions in light of studies indicating that IL levels in human PTSD populations tend to decrease over time, even as PTSD symptoms persist (Toft et al., 2022). This apparent contradiction between animal and human studies may indicate a more complex temporal and species-specific pattern of immune regulation, suggesting that while IL-mediated B-cell activation plays a role in acute PTSD, it may diminish with chronic onset. IL-mediated responses may result from immune system shifts, long-term memory encoding, or epigenetic modifications. This is supported by research showing that longitudinal oxidative stress significantly and longitudinally impacts telomere length (Minelli et al., 2025; Zhang et al., 2025).

Pathway analyses also revealed intermediary connections between B-cell function and PTSD remission, with specific gene loci and vectors linking immune function to psychological outcomes. These pathways provide a mechanistic bridge that may explain how immune modulation can impact remission and therapeutic efficacy. Notably, this analysis mapped interactions among B-cell function, PTSD remission, and the adaptive immune response in precise detail, suggesting that B-cell-mediated immune responses could be integral to the underlying biological processes in both PTSD and EMDR.

These findings lend support to models that view PTSD not solely as a psychological phenomenon but as one rooted in immune response, inflammatory processes, and neuronal morphology, thus reinforcing the potential for integrated non-invasive treatments targeting both psychological and biological factors simultaneously. Further investigation into the longitudinal effects of IL-10 and related cytokines is warranted to clarify their roles in the trajectory of PTSD and progression of EMDR mechanisms.

### 4.5 Implications for bilateral stimulation and EMDR mechanisms

In addition to IL-10’s established role, this analysis uncovered critical intermediary pathways and gene loci that directly link B-cell function to PTSD remission, notably through IL-10 receptor epigenetic mechanisms. This finding aligns with recent research suggesting that epigenetic modifications, particularly those affecting immune receptor function, may facilitate long-term changes in immune function and memory formation, both of which are implicated in PTSD and its treatment through EMDR (Provençal and Binder, 2015).

This analysis supports the hypothesis that EMDR may leverage epigenetic neuronal and proteomic immune mechanisms to facilitate symptom remission, potentially by modulating inflammatory cytokines such as IL-6, IL-10, and NPY, all of which are integral to the PTSD inflammatory response (de Oliveira et al., 2018).

The discovery of these potential intermediary immune pathways has significant implications for understanding EMDR and the mechanisms underlying bilateral treatments. By mapping the interactions between immune function and PTSD remission, this study supports a multivariate model of EMDR, suggesting that bilateral stimulation may modulate immune responses in addition to memory processing. These findings align with a tripartite model suggestion that EMDR works through neurobiological, epigenetic, and immune-mediated pathways simultaneously, potentially via mechanisms that enhance IL-10 activity and promote remission (de Oliveira et al., 2018; Tabibnia, 2020; Dell’Oste et al., 2023).

Given that IL-10 and related cytokines are implicated in inflammation and immune regulation, it is plausible that bilateral stimulation contributes to EMDR efficacy by catalyzing immune down-regulation, thereby mitigating PTSD-related inflammatory responses. This connection suggests that EMDR efficacy may involve an immune-mediated pathway that intersects with multivariate and AIP models of PTSD, potentially providing a more comprehensive neurobiological framework for understanding behavioral outcomes.

### 4.6 Limitations

While these findings offer valuable insights into the immune and genetic functionality involved in PTSD remission and EMDR efficacy, limitations exist. Differences between IL activation in animal models and human studies underscore the need for further experimental research to understand immune responses over different PTSD stages and demographics. Exploring additional potential epigenetic and proteomic contributors beyond IL-10 and B-cell functionality could provide a more comprehensive understanding of EMDR’s biological underpinnings.

An additional limitation of this study was the inability to examine apoptosis alongside B-cell differentiation and proliferation, specifically in terms of Z-DNA morphology, telomere elongation, protein misfolding, and DMN dysregulation. Incorporating this B-cell subclass and neuronal subtypes into the analysis would further inform the potential prominence of IL-10 in memory formation, consolidation, inflammation, and synaptic depotentiation for PTSD remission and associated therapies. Combining IL-6, IL-10, NPY, and OXY, along with additional genes related to both EMDR functionality and PTSD remission in the context of apoptosis, may also provide insight into proteomic function (Arntzen and Thiede, 2012; Lynall et al., 2021; Waszczuk et al., 2023).

Alongside this, the inability to explore the functionality and contributions of Z-DNA morphology and telomere augmentation in this analysis was a limitation. Incorporating higher-order rotational vectors, recursive permutations, Mendelian randomization, high-density microelectrode array data, and iPSC-derived cell lines may reveal specific neuronal functions to uncover active mechanisms in light-based therapies, thereby resolving conflicting experimental findings and ultimately reconciling current debates regarding applications.

### 4.7 Strengths and future perspectives

This study demonstrated that enhanced computing power, leveraging of nonlinear AI models, incorporating temporal dimensionality and chaotic parameters, and access to optimized server clusters improved the robustness of all analyses, addressing prior computational limitations in behavioral neuroscience. Technological improvements support the feasibility of similar analyses to reconcile debate regarding PTSD functionality and EMDR efficacy, while also reducing relapse rates in targeted therapeutics. Supplementing findings with high-density microelectrode array data and iPSC-derived neurons will provide additional insights into neuronal function during targeted treatments, distinguishing between myelinated and unmyelinated axons, and shedding light on telomere shortening observed in longitudinal studies of PTSD (Minelli et al., 2025; Zhang et al., 2025).

Inclusion of transcriptomic and genomic data from human, mammalian, cell-line, organoid, and animal samples represents a substantial advance in understanding both PTSD and EMDR. By examining epigenetic and proteomic functions, we move toward a more cohesive model of PTSD, EMDR, and associated treatment efficacy. Future studies should expand on these findings using *in vivo* clinical monitoring and genetic sequencing in both clinical and nonclinical populations for comparison. Isolating the active neurobiological mechanisms underlying bilateral stimuli will help clarify how hemispheric manipulation influences cognitive shifts, genetic modifications, and epigenetic changes.

Additional studies exploring the systemic impacts of NPY binding domains and protein folding orientations are anticipated for the following research phase. Further studies will also confirm whether OXY findings are species- or cell line-specific and explore how PACAP affects OXY-facilitated therapeutic outcomes. Including biometric data into this experimental protocol will facilitate a better understanding of the neurobiological mechanisms in PTSD, cellular functionality, and locality of associated memory formation, and molecular underpinnings of EMDR.

By testing varying bilateral exposure times and rates while monitoring response types, target cognitive domains can be better assessed to evaluate changes in IL-6, IL-10, NPY, OXY, PACAP, and ACSS2 levels. Insights from simultaneous fMRI imaging, EEG monitoring, and serial electron microscopy will provide additional precision in measuring inter- and intra-hemispheric shifts during EMDR administration, potentially clarifying optimal timing and exposure, DMN activation and engagement, and nature of hemispheric interactions (Tinsley et al., 2016). Differential proteomic analyses of the glymphatic system may further elucidate the roles of inflammation and apoptosis in PTSD, thereby aiding in determining the dominant model guiding bilateral efficacy through explicit examination of how up- and down-regulation occur. Systematic characterization of DMN priming may help identify additional mechanisms underlying therapeutic efficacy.

Exploring protein “death folding” mechanisms and Z-DNA morphology may provide additional insights into CNS glutamate atrophy in neurons. This research may elucidate why and how PTSD exposure contributes to secondary onset of comorbid progressive diseases such as ALS, Parkinson’s, and Alzheimer’s. Integrating human cohorts with iPSC-derived neurons for analysis may help identify the precise DNA loci associated with bilateral therapies, thereby improving our understanding of symptom onset and targeted therapeutic strategies.

Longitudinal studies assessing the temporal stability of bilateral exposure, in conjunction with the neuronal effects of EMDR on memory, are planned to rule out potential confounding factors. Insights from such research can help address the 30% minimum PTSD relapse rate currently observed (Peterson et al., 2017; Berge et al., 2020; Brooks and Greenberg, 2024). By incorporating inflammatory response and B-cell functional data, future research may provide critical perspectives on mitigating PTSD onset and enhancing therapeutic efficacy through refinement of existing treatment models, as well as clarification on individual variations in EMDR outcomes across different cohorts and trauma types.

## 5 Conclusion

Exploring behavioral neuroscience, epigenetics, and proteomics via dynamic pathway analysis and systemic Monte Carlo Methods enhances experimental findings through increased predictive power and generalizability. This approach better supports target populations by revealing previously hidden factors and underlying mechanisms.

Using this novel methodology, this study confirms an association between B-cell function, interleukin signaling (IL-6, IL-10), NPY, and PTSD remission. Monte Carlo simulation supported IL-10 as a central nexus between systems and NPY as a critical therapeutic target, suggesting that immune function and neuropeptide activity play a key role in EMDR efficacy and PTSD resilience. Immunological shifts observed after intervention highlight a potential link between innate immune response and adaptive treatment outcomes. Variability in immune response may also explain EMDR relapse and failure rates, offering new directions for personalized PTSD treatment via neuronal mechanisms.

The findings suggest that EMDR influences neuroimmune pathways through recalibration of the DMN, hemispheric integration, and reduction of allostatic load. Moreover, Dopaminergic and Histaminergic signaling, HPA axis modulation, and epigenetic factors may also contribute to long-term remission from PTSD. These results underscore the need for integrating neuroimmune mechanisms and epigenetic regulation into PTSD treatments to optimize EMDR efficacy and reduce relapse rates. Specifically, active targeting of IL-10 within the first year of trauma exposure and symptom onset, or IL-6 within the 2 weeks of adversity exposure, may significantly and positively impact PTSD outcomes. This may also significantly increase EMDR efficacy and impact (Ting et al., 2020; Toft et al., 2022; Kosanovic Rajacic et al., 2025).

## Data Availability

The datasets presented in this study can be found in online repositories. The names of the repository/repositories and accession number(s) can be found in the article/Supplementary material.

## Footnotes

1
https://www.informatics.jax.org/

2 GeneMANIA^®^ software: https://genemania.org/

## References

[B1] Adams E. J. Nguyen A. T. Cowan N. (2018). Theories of working memory: Differences in definition, degree of modularity, role of attention, and purpose. *Lang. Speech Hear. Serv. Sch.* 49 340–355. 10.1044/2018_LSHSS-17-0114 29978205 PMC6105130

[B2] Adams R. Ohlsen S. Wood E. (2020). Eye Movement Desensitization and Reprocessing (EMDR) for the treatment of psychosis: A systematic review. *Eur. J. Psychotraumatol.* 11:1711349. 10.1080/20008198.2019.1711349 32284817 PMC7144286

[B3] Akiki T. J. Averill C. L. Wrocklage K. M. Scott J. C. Averill L. A. Schweinsburg B. et al. (2018). Default mode network abnormalities in posttraumatic stress disorder: A novel network-restricted topology approach. *Neuroimage* 176 489–498. 10.1016/J.NEUROIMAGE.2018.05.005 29730491 PMC5976548

[B4] Alexander D. C. Corman T. Mendoza M. Glass A. Belity T. Wu R. et al. (2022). Targeting acetyl-CoA metabolism attenuates the formation of fear memories through reduced activity-dependent histone acetylation. *Proc. Natl. Acad. Sci. U. S. A.* 119:e2114758119. 10.1073/pnas.2114758119 35921439 PMC9371679

[B5] Arntzen M. Thiede B. (2012). ApoptoProteomics, an integrated database for analysis of proteomics data obtained from apoptotic cells. *Mol. Cell. Proteomics* 11:10447. 10.1074/mcp.M111.010447 22067098 PMC3277752

[B6] Baddeley A. D. Hitch G. (1974). Working memory. *Psychol. Learn. Motiv. Adv. Res. Theory* 8 47–89. 10.1016/S0079-7421(08)60452-1

[B7] Berendzen K. M. Sharma R. Mandujano M. A. Wei Y. Rogers F. D. Simmons T. C. et al. (2023). Oxytocin receptor is not required for social attachment in prairie voles. *Neuron* 111 787–796.e4. 10.1016/J.NEURON.2022.12.011 36708707 PMC10150797

[B8] Berge E. E. Hagen R. Øveraas Halvorsen J. (2020). PTSD relapse in veterans of Iraq and Afghanistan: A systematic review. *Military Psychol.* 32 300–312. 10.1080/08995605.2020.1754123 38536379 PMC10013559

[B9] Bountress K. E. Bacanu S. A. Tomko R. L. Korte K. J. Hicks T. Sheerin C. et al. (2018). The effects of a BDNF Val66Met polymorphism on posttraumatic stress disorder: A meta-analysis. *Neuropsychobiology* 76:136. 10.1159/000489407 29874672 PMC6057796

[B10] Bowers M. E. Choi D. C. Ressler K. J. (2012). Neuropeptide regulation of fear and anxiety: Implications of cholecystokinin, endogenous opioids, and neuropeptide Y. *Physiol. Behav.* 107 699–710. 10.1016/j.physbeh.2012.03.004 22429904 PMC3532931

[B11] Brenna A. Olejniczak I. Chavan R. Ripperger J. A. Langmesser S. Cameroni E. et al. (2019). Cyclin dependent kinase 5 (Cdk5) regulates the circadian clock. *Elife* 8:e50925. 10.7554/ELIFE.50925 31687929 PMC6890458

[B12] Brooks S. K. Greenberg N. (2024). Recurrence of post-traumatic stress disorder: Systematicreview of definitions, prevalence and predictors. *BMC Psychiatry* 24:37. 10.1186/S12888-023-05460-X 38195482 PMC10777598

[B13] Carmassi C. Marazziti D. Mucci F. Della Vecchia A. Barberi F. M. Baroni S. et al. (2021). Decreased plasma oxytocin levels in patients with PTSD. *Front. Psychol.* 12:612338. 10.3389/fpsyg.2021.612338 34276462 PMC8280334

[B14] Carvalho Silva R. Martini P. Hohoff C. Mattevi S. Bortolomasi M. Menesello V. et al. (2024). DNA methylation changes in association with trauma-focused psychotherapy efficacy in treatment-resistant depression patients: A prospective longitudinal study. *Eur. J. Psychotraumatol.* 15:2314913. 10.1080/20008066.2024.2314913 38362742 PMC10878335

[B15] de Oliveira J. F. Wiener C. D. Jansen K. Portela L. V. Lara D. R. Souza L. D. et al. (2018). Serum levels of interleukins IL-6 and IL-10 in individuals with posttraumatic stress disorder in a population-based sample. *Psychiatry Res.* 260 111–115. 10.1016/J.PSYCHRES.2017.11.061 29179015

[B16] de Voogd L. D. Kanen J. W. Neville D. A. Roelofs K. Fernández G. Hermans E. J. (2018). Eye-movement intervention enhances extinction via amygdala deactivation. *J. Neurosci.* 38 8694–8706. 10.1523/JNEUROSCI.0703-18.2018 30181134 PMC6596227

[B17] Dell’Oste V. Fantasia S. Gravina D. Palego L. Betti L. Dell’Osso L. et al. (2023). Metabolic and inflammatory response in Post-traumatic stress disorder (PTSD): A systematic review on peripheral neuroimmune biomarkers. *Int. J. Environ. Res. Public Health* 20:2937. 10.3390/IJERPH20042937 36833633 PMC9957545

[B18] Difede J. Olden M. Cukor J. (2014). Evidence-based treatment of post-traumatic stress disorder. *Annu. Rev. Med.* 65 319–332. 10.1146/ANNUREV-MED-051812-145438 24422573

[B19] Feder A. Nestler E. J. Charney D. S. (2009). Psychobiology and molecular genetics of resilience. *Nat. Rev. Neurosci.* 10:446. 10.1038/NRN2649 19455174 PMC2833107

[B20] Flanagan J. C. Mitchell J. M. (2019). Augmenting treatment for posttraumatic stress disorder and co-occurring conditions with oxytocin. *Curr. Treat. Options Psychiatry* 6:132. 10.1007/S40501-019-00171-1 31763133 PMC6874414

[B21] Fluckiger A. C. Garrone P. Durand I. Galizzi J. P. Banchereau J. (1993). Interleukin 10 (IL-10) upregulates functional high affinity IL-2 receptors on normal and leukemic B lymphocytes. *J. Exp. Med.* 178 1473–1481. 10.1084/JEM.178.5.1473 8228801 PMC2191252

[B22] Franz M. Rodriguez H. Lopes C. Zuberi K. Montojo J. Bader G. D. et al. (2018). GeneMANIA update 2018. *Nucleic Acids Res.* 46 W60–W64. 10.1093/NAR/GKY311 29912392 PMC6030815

[B23] Furukawa K. Ohmi Y. Yesmin F. Tajima O. Kondo Y. Zhang P. et al. (2020). Novel molecular mechanisms of gangliosides in the nervous system elucidated by genetic engineering. *Int. J. Mol. Sci.* 21:1906. 10.3390/IJMS21061906 32168753 PMC7139306

[B24] Gibbs A. A. Bautista C. E. Mowlem F. D. Naudts K. H. Duka D. T. (2014). Catechol-O- methyltransferase val158met genotype determines effect of reboxetine on emotional memory in healthy male volunteers. *J. Psychiatry Neurosci.* 39:E24. 10.1503/JPN.130131 24467942 PMC3997609

[B25] Golubeva E. Zeltser A. Zorkina Y. Ochneva A. Tsurina A. Andreyuk D. et al. (2024). Epigenetic alterations in post-traumatic stress disorder: Comprehensive review of molecular markers. *Complex Psychiatry* 10 71–107. 10.1159/000541822 39564465 PMC11573359

[B26] Govindula A. Ranadive N. Nampoothiri M. Rao C. M. Arora D. Mudgal J. (2023). Emphasizing the crosstalk between inflammatory and neural signaling in post-traumatic stress disorder (PTSD). *J. Neuroimmune Pharmacol.* 18 248–266. 10.1007/S11481-023-10064-Z 37097603 PMC10577110

[B27] Hase M. Balmaceda U. M. Ostacoli L. Liebermann P. Hofmann A. (2017). The AIP model of EMDR therapy and pathogenic memories. *Front. Psychol.* 8:1578. 10.3389/FPSYG.2017.01578 28983265 PMC5613256

[B28] Hwang A. Skarica M. Xu S. Coudriet J. Lee C. Y. Lin L. et al. (2025). Single-cell transcriptomic and chromatin dynamics of the human brain in PTSD. *Nature* 643 744–754. 10.1038/s41586-025-09083-y 40533550 PMC12267058

[B29] Jonas D. E. Cusack K. Forneris C. A. Wilkins T. M. Sonis J. Middleton J. C. et al. (2013). Psychological and pharmacological treatments for adults with Posttraumatic stress disorder (PTSD). *Comp. Effect. Rev.* 760 21–118. 10.23970/AHRQEPCCER207 23658937

[B30] Katrinli S. Oliveira N. C. S. Felger J. C. Michopoulos V. Smith A. K. (2022). The role of the immune system in posttraumatic stress disorder. *Transl. Psychiatry* 12 1–14. 10.1038/s41398-022-02094-7 35927237 PMC9352784

[B31] Keaton S. A. Arnetz J. Jamil H. Dhalimi A. Stemmer P. M. Ruden D. M. et al. (2021). IL-10: A possible immunobiological component of positive mental health in refugees. *Compr. Psychoneuroendocrinol.* 8:100097. 10.1016/J.CPNEC.2021.100097 35757662 PMC9216633

[B32] Kim T. D. Lee S. Yoon S. (2020). Inflammation in Post-traumatic stress disorder (PTSD): A review of potential correlates of PTSD with a neurological perspective. *Antioxidants* 9:107. 10.3390/ANTIOX9020107 31991875 PMC7070581

[B33] Kitamura T. Ogawa S. K. Roy D. S. Okuyama T. Morrissey M. D. Smith L. M. et al. (2017). Engrams and circuits crucial for systems consolidation of a memory. *Science* 356 73–78. 10.1126/science.AAM6808 28386011 PMC5493329

[B34] Kosanovic Rajacic B. Sagud M. Begic D. Nikolac Perkovic M. Kozmar A. Rogic D. et al. (2025). Increased interleukin-6 levels in responders with treatment-resistant depression after bright light therapy. *Biomolecules* 15:295. 10.3390/biom15020295 40001598 PMC11852636

[B35] Kuan P. F. Yang X. Ren X. Che C. Waszczuk M. Kotov R. et al. (2021). Mapping the transcriptomics landscape of post-traumatic stress disorder symptom dimensions in World Trade Center responders. *Transl. Psychiatry* 11:310. 10.1038/S41398-021-01431-6 34031375 PMC8144574

[B36] Landin-Romero R. Novo P. Vicens V. McKenna P. J. Santed A. Pomarol-Clotet E. et al. (2013). EMDR therapy modulates the default mode network in a subsyndromal, traumatized bipolar patient. *Neuropsychobiology* 67 181–184. 10.1159/000346654 23548794

[B37] Laure Wadji D. Martin-Soelch C. Camos V. (2022). Can working memory account for EMDR efficacy in PTSD? *BMC Psychol.* 10:245. 10.1186/s40359-022-00951-0 36320044 PMC9623920

[B38] Lawrence S. Scofield R. H. (2024). Post traumatic stress disorder associated hypothalamic- pituitary-adrenal axis dysregulation and physical illness. *Brain Behav. Immun. Health* 41:100849. 10.1016/J.BBIH.2024.100849 39280087 PMC11401111

[B39] Le Dorze C. Borreca A. Pignataro A. Ammassari-Teule M. Gisquet-Verrier P. (2020). Emotional remodeling with oxytocin durably rescues trauma-induced behavioral and neuro- morphological changes in rats: A promising treatment for PTSD. *Transl. Psychiatry* 10:27. 10.1038/S41398-020-0714-0 32066681 PMC7026036

[B40] Li Q. Bartley A. F. Dobrunz L. E. (2017). Endogenously released neuropeptide Y suppresses hippocampal short-term facilitation and is impaired by stress-induced anxiety. *J. Neurosci.* 37 23–37. 10.1523/JNEUROSCI.2599-16.2016 28053027 PMC5214634

[B41] Liang M. Mouraux A. Hu L. Iannetti G. D. (2013). Primary sensory cortices contain distinguishable spatial patterns of activity for each sense. *Nat. Commun.* 4:1979. 10.1038/ncomms2979 23752667 PMC3709474

[B42] Lynall M. E. Kigar S. L. Lehmann M. L. DePuyt A. E. Tuong Z. K. Listwak S. J. et al. (2021). B-cells are abnormal in psychosocial stress and regulate meningeal myeloid cell activation. *Brain Behav. Immun.* 97 226–238. 10.1016/j.bbi.2021.08.002 34371135 PMC8453122

[B43] Marshall P. R. Zhao Q. Li X. Wei W. Periyakaruppiah A. Zajaczkowski E. L. et al. (2020). Dynamic regulation of Z-DNA in the mouse prefrontal cortex by the RNA editing enzyme Adar1 is required for fear extinction. *Nat. Neurosci.* 23:718. 10.1038/S41593-020-0627-5 32367065 PMC7269834

[B44] Matthijssen S. J. M. A. Verhoeven L. C. M. van den Hout M. A. Heitland I. (2017). Auditory and visual memories in PTSD patients targeted with eye movements and counting: The effect of modality-specific loading of working memory. *Front. Psychol.* 8:286723. 10.3389/fpsyg.2017.01937 PMC5675874 29163311

[B45] Maul S. Giegling I. Fabbri C. Corponi F. Serretti A. Rujescu D. (2020). Genetics of resilience: Implications from genome-wide association studies and candidate genes of the stress response system in posttraumatic stress disorder and depression. *Am. J. Med. Genet. B Neuropsychiatr. Genet.* 183 77–94. 10.1002/AJMG.B.32763 31583809

[B46] Maxfield L. Melnyk W. T. Hayman G. C. A. (2008). A working memory explanation for the effects of eye movements in EMDR. *J. EMDR Pract. Res.* 2 247–261. 10.1891/1933-3196.2.4.247

[B47] Mews P. Donahue G. Drake A. M. Luczak V. Abel T. Berger S. L. (2017). Acetyl-CoA synthetase regulates histone acetylation and hippocampal memory. *Nature* 546 381–386. 10.1038/nature22405 28562591 PMC5505514

[B48] Minelli A. Meloni A. Bortolomasi M. Pisanu C. Zampieri E. Congiu D. et al. (2025). Telomere length and mitochondrial DNA copy number in association with trauma-focused psychotherapy efficacy. *Neurosci. Appl.* 4:104095. 10.1016/J.NSA.2024.104095 40654587 PMC12244152

[B49] Mostafavi S. Ray D. Warde-Farley D. Grouios C. Morris Q. (2008). GeneMANIA: A real-time multiple association network integration algorithm for predicting gene function. *Genome Biol.* 9 1–15. 10.1186/gb-2008-9-s1-s4 PMC2447538 18613948

[B50] Mustafa M. Ahmad R. Tantry I. Q. Ahmad W. Siddiqui S. Alam M. et al. (2024). Apoptosis: A comprehensive overview of signaling pathways, morphological changes, and physiological significance and therapeutic implications. *Cells* 13:1838. 10.3390/CELLS13221838 39594587 PMC11592877

[B51] Neylan T. C. O’Donovan A. (2019). *Inflammation and PTSD.* San Francisco, CA: National Center for PTSD. 10.1001/jamapsychiatry.2013.4374

[B52] Nievergelt C. M. Maihofer A. X. Klengel T. Atkinson E. G. Chen C. Y. Choi K. W. et al. (2019). International meta-analysis of PTSD genome-wide association studies identifies sex- and ancestry-specific genetic risk loci. *Nat. Commun.* 10 1–16. 10.1038/s41467-019-12576-w 31594949 PMC6783435

[B53] Nievergelt C. M. Maihofer A. X. Mustapic M. Yurgil K. A. Schork N. J. Miller M. W. et al. (2015). Genomic predictors of combat stress vulnerability and resilience in U.S. Marines: A genome-wide association study across multiple ancestries implicates PRTFDC1 as a potential PTSD gene. *Psychoneuroendocrinology* 51 459–471. 10.1016/J.PSYNEUEN.2014.10.017 25456346

[B54] Niitsu K. Rice M. J. Houfek J. F. Stoltenberg S. F. Kupzyk K. A. Barron C. R. (2019). A systematic review of genetic influence on psychological resilience. *Biol. Res. Nurs.* 21 61–71. 10.1177/1099800418800396 30223673

[B55] Olff M. (2017). Sex and gender differences in post-traumatic stress disorder: An update. *Eur. J. Psychotraumatol.* 8:4. 10.1080/20008198.2017.1351204

[B56] Parrish R. R. Gupta S. Lubin F. D. (2010). The epigenetics of memory storage in the brain. *Cellscience* 7 1–8. PMC8439273 34527072

[B57] Peterson K. Bourne D. Anderson J. Mackey K. Helfand M. (2017). *Evidence Brief: Effectiveness of Stellate Ganglion Block for Treatment of Posttraumatic Stress Disorder (PTSD).* Washington, DC: Department of Veterans Affairs. 28742302

[B58] Provençal N. Binder E. B. (2015). The effects of early life stress on the epigenome: From the womb to adulthood and even before. *Exp. Neurol.* 268 10–20. 10.1016/J.EXPNEUROL.2014.09.001 25218020

[B59] Reichmann F. Holzer P. (2016). Neuropeptide Y: A stressful review. *Neuropeptides* 55 99–109. 10.1016/J.NPEP.2015.09.008 26441327 PMC4830398

[B60] Renner V. Joraschky P. Kirschbaum C. Schellong J. Petrowski K. (2022). Pro- and anti-inflammatory cytokines Interleukin-6 and Interleukin-10 predict therapy outcome of female patients with posttraumatic stress disorder. *Transl. Psychiatry* 12:472. 10.1038/s41398-022-02230-3 36351891 PMC9646837

[B61] Sack M. Spieler D. Wizelman L. Epple G. Stich J. Zaba M. et al. (2017). Intranasal oxytocin reduces provoked symptoms in female patients with posttraumatic stress disorder despite exerting sympathomimetic and positive chronotropic effects in a randomized controlled trial. *BMC Med.* 2017:40. 10.1186/S12916-017-0801-0 28209155 PMC5314583

[B62] Shapiro F. (2018). *Eye Movement Desensitization and Reprocessing (EMDR) Therapy: Basic Principles, Protocols, and Procedures*, 3rd Edn. New York, NY: The Guilford Press.

[B63] Speakman S. White K. LaPorta A. J. Payton M. E. Gubler K. D. Ryznar R. J. (2023). Cytokine fluctuation during acute stress is correlated to life trauma. *J. Trauma Acute Care Surg.* 95 535–541. 10.1097/TA.0000000000004006 37165473 PMC10545070

[B64] Stanic B. Van De Veen W. Wirz O. F. Rückert B. Morita H. Söllner S. et al. (2015). IL-10-overexpressing B cells regulate innate and adaptive immune responses. *J. Allergy Clin. Immunol.* 135 771–780.e8. 10.1016/j.jaci.2014.07.041 25240783

[B65] Stickgold R. (2002). EMDR: A putative neurobiological mechanism of action. *J. Clin. Psychol.* 58 61–75. 10.1002/JCLP.1129 11748597

[B66] Tabibnia G. (2020). An affective neuroscience model of boosting resilience in adults. *Neurosci. Biobehav. Rev.* 115 321–350. 10.1016/J.NEUBIOREV.2020.05.005 32522489

[B67] Ting E. Y. C. Yang A. C. Tsai S. J. (2020). Role of Interleukin-6 in depressive disorder. *Int. J. Mol. Sci.* 21:2194. 10.3390/ijms21062194 32235786 PMC7139933

[B68] Tinsley J. N. Molodtsov M. I. Prevedel R. Wartmann D. Espigulé-Pons J. Lauwers M. et al. (2016). Direct detection of a single photon by humans. *Nat. Commun.* 7:12172. 10.1038/ncomms12172 27434854 PMC4960318

[B69] Toft H. Bramness J. G. Lien L. (2022). Levels of peripheral circulating IL-6 and IL-10 decrease over time despite high depression burden in PTSD patients. *Neuropsychiatr. Dis. Treat.* 18 737–747. 10.2147/NDT.S357797 35414745 PMC8995001

[B70] Van Den Hout M. A. Engelhard I. M. Beetsma D. Slofstra C. Hornsveld H. Houtveen J. et al. (2011). EMDR and mindfulness. Eye movements and attentional breathing tax working memory and reduce vividness and emotionality of aversive ideation. *J. Behav. Ther. Exp. Psychiatry* 42 423–431. 10.1016/J.JBTEP.2011.03.004 21570931

[B71] Vinkers C. H. Geuze E. van Rooij S. J. H. Kennis M. Schür R. R. Nispeling D. M. et al. (2021). Successful treatment of post-traumatic stress disorder reverses DNA methylation marks. *Mol. Psychiatry* 26 1264–1271. 10.1038/s41380-019-0549-3 31645664

[B72] Wang X. Krieger-Redwood K. Cui Y. Smallwood J. Du Y. Jefferies E. (2024). Macroscale brain states support the control of semantic cognition. *Commun. Biol.* 2024:926. 10.1038/s42003-024-06630-7 39090387 PMC11294576

[B73] Waszczuk M. A. Kuan P. F. Yang X. Miao J. Kotov R. Luft B. J. (2023). Discovery and replication of blood-based proteomic signature of PTSD in 9/11 responders. *Transl. Psychiatry* 13:8. 10.1038/S41398-022-02302-4 36631443 PMC9834302

[B74] Williams S. Ralph M. L. Jung J. (2023). The role of GABA in semantic memory and its neuroplasticity. *Elife* 12 1–16. 10.7554/ELIFE.91771.1 PMC12133155 40459014

[B75] Witteveen A. B. Stramrood C. A. Henrichs J. Flanagan J. C. van Pampus M. G. Olff M. et al. (2019). The oxytocinergic system in PTSD following traumatic childbirth: Endogenous and exogenous oxytocin in the peripartum period. *Arch. Women’s Mental Health* 23 317–329. 10.1007/S00737-019-00994-0 31385103 PMC7244459

[B76] Wu G. Feder A. Cohen H. Kim J. J. Calderon S. Charney D. S. et al. (2013). Understanding resilience. *Front. Behav. Neurosci.* 7:10. 10.3389/FNBEH.2013.00010 23422934 PMC3573269

[B77] Zhang H. Zhou J. Cao Y. Zhang X. Chang H. Zhao Y. et al. (2025). Association between telomere length and psychiatric disorders: A bidirectional Mendelian randomization study. *Eur. Arch. Psychiatry Clin. Neurosci.* 276 1959–1966. 10.1007/S00406-025-02008-W 40278882

