# Supplementary material for: Study on the cognitive effects of eye movement desensitization and reprocessing: proposed active mechanisms
Source: Front Comput Neurosci. 2026 Aug 10;20:1751744. doi: 10.3389/fncom.2026.1751744 (PMC13500570; doi:10.3389/fncom.2026.1751744)
Supplement: Supplementary file 1 [file Data_Sheet_1.pdf]

PSTD-associated Genes

PSTD Remission	*PTSD Onset*			
	Avoidance	*Re-experiencing*	*Numbing*	*Hyperarousal*
5-HTT	AHSP	SIGLEC10	ATP2A3	~~LOC728743~~ ->
SERT1	ALAS2	UPF2	HAUS4	STMN3
ACSS2	CA1	POLD3	RNPS1	
APOE	CCNDBP1		RAD51B	
BDNF	CMAS			
CACNA1C	CREG1			
CDK5	CTNNAL1			
COMT	DPCD			
CRHBP	GABARAPL2			
CRHR1	GLRX5			
DAT	H2AFJ			
DRD2	HBD			
DRD3	HMBS			
DRD4	PAGE2B			
FKBP5	PCTP			
IL10	PITHD1			
IL6	POLR1D			
IL6R	PRDX2			
IL6ST	RGCC			
LMO3	RRAGA			
MAOA	SLC22A4			
MTHFR	TERF2IP			
NPY	TFRC			
OPRM1				
OXT				
OXTR				
RNASE13				
SLC6A3				
SLC6A3				
SLC6A4				
STXBP5L				
TNFa				
TNFR1				
TNFR2				

B-cell associated Genes

Upregulation of B cell	
Differentiation	***Proliferation***
ATP11C	ADA
BAD	ATAD5
CD27	BCL2
IL2	BCL6
IL2RG	BMI1
IL7	BST1
IL10	BTK
IL21	CARD11
INPP5D	CD38
MMP14	CD40
NCKAP1L	CD40LG
PCID2	CD74
PNP	CD81
PPP2R3C	CD320
PRDM1	CDKN1A
SPI1	CHRNB2
STAT5A	CLCF1
STAT5B	EPHB2
SYK	GPR183
XBP1	IL2
	IL3
	IL4
	IL5
	IL7
	IL13
	IL21
	IRS2
	MEF2C
	MIF
	NCKAP1L
	NFATC2
	PELI1
	PRLR
	PTPRC
	SASH3
	SLC39A10
	TCF3
	TFRC
	TICAM1
	TIRAP
	TLR4
	TLR9

TNFRSF4
TNFRSF13C
TNFSF13B
VAV3
WNT3A

B-cell associated Genes	
Downregulation of B-cell	
Differentiation	*Proliferation*
BCL6	ATM
FLT3	BTK
HMGB3	BTLA
ID2	CASP3
SFRP1	CD24A
	CD300A
	CDKN2A
	CTLA4
	FCGR2B
	IL10
	INPP5D
	LYN
	PAWR
	PKN1
	PRDM1
	PTEN
	RC3H1
	TNFRSF13B
	TNFRSF21
	TSC2
	TYROBP

B-cell associated Genes
Apoptosis Up & Down Regulation

AIFM1	IRAK4
AKT1	MAP3K14
AKT2	MYD88
AKT3	NFKB1
APAF1	NFKB2
ATM	NFKBIA
BAD	NTRK1
BAX	PIK3CA
BCL2	PIK3CB
BCL2L1	PIK3CD
BID	PIK3CG
BIRC2	PIK3R1
BIRC3	PIK3R2
CAPN1	PIK3R3
CAPN2	PIK3R5
CASP10	PPP3CA
CASP3	PPP3CB
CASP6	PPP3CC
CASP7	PPP3R1
CASP8	PPP3R2
CASP9	PRKACA
CFLAR	PRKACB
CHUK	PRKACG
CSF2RB	PRKAR1A
CYCS	PRKAR1B
DFFA	PRKAR2A
DFFB	PRKAR2B
ENDOG	RELA
FADD	RIPK1
FAS	TNF
FASLG	TNFRSF10A
IKBKB	TNFRSF10B
IKBKG	TNFRSF10C
IL1A	TNFRSF10D
IL1B	TNFRSF1A
IL1R1	TNFSF10
IL1RAP	TP53
IL3	TRADD
IL3RA	TRAF2
IRAK1	
IRAK2	
IRAK3	
