# Supplementary material for: Study on the cognitive effects of eye movement desensitization and reprocessing: proposed active mechanisms
Source: Front Comput Neurosci. 2026 Aug 10;20:1751744. doi: 10.3389/fncom.2026.1751744 (PMC13500570; doi:10.3389/fncom.2026.1751744)
Supplement: Supplementary file 4 [file Data_Sheet_4.pdf]

# GeneMANIA report

Created on : 23 May 2023 11:17:07
Last database update : 13 August 2021 00:00:00
Application version : 3.6.0

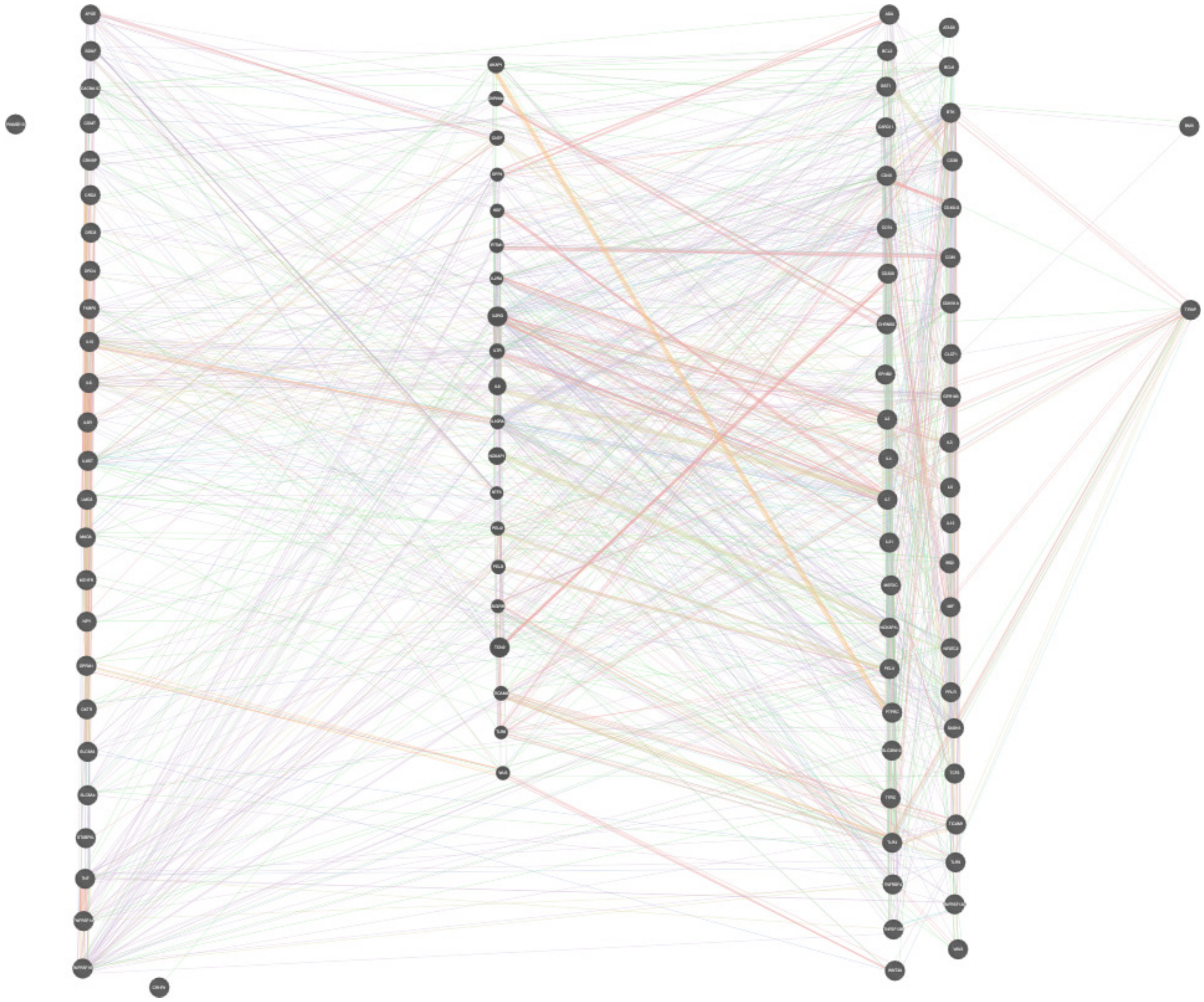

## Networks

- Co-expression
- Physical Interactions
- Predicted
- Co-localization
- Shared protein domains
- Pathway
- Genetic Interactions

## Functions

N/A

# Search parameters

**Organism** Homo sapiens (human)

**Genes** CD38 , BTK , CD74 , LMO3 , DRD4 , TCF3 , TFRC , MEF2C , PTPRC , COMT , FKBP5 , CD40 , NFATC2 , CD40LG , TNFSF13B , IL7 , SLC6A4 , IL2 , BST1 , CD81 , OPRM1 , PRLR , IL4 , IL5 , BCL6 , CRHR1 , SASH3 , NPY , NCKAP1L , CDKN1A , TICAM1 , APOE , EPHB2 , VAV3 , IL6ST , IL6 , IL10 , TLR4 , IL21 , SLC6A3 , STXBP5L , CRHBP , DRD2 , TIRAP , CACNA1C , DRD3 , WNT3A , TNFRSF13C , IL6R , CHRNB2 , IL3 , CD320 , BMI1 , IL13 , GPR183 , BCL2 , CLCF1 , ATAD5 , BDNF , MTHFR , OXTR , IRS2 , TNFRSF4 , MAOA , ADA , SLC39A10 , PELI1 , CARD11 , RNASE13 , TLR9 , MIF , TNFR2 , TNFR1 , 5-HTT , SERT1 , DAT , TNFA

**Network weighting** Automatically selected weighting method

**Networks**

**A**

---

Abbasi-Schild-Poulter-2019 , Abu-Odeh-Aqeilan-2014 , Achuthankutty-Mailand-2019 , Agrawal-Sedivy-2010 , Ahn-Lee-2008 , Albers-Koegl-2005 , Alexander-Wang-2018 , Alexandru-Deshaies-2008 , Alizadeh-Staudt-2000 , Alsulami-Cagney-2019 , An-Sun-2017 , Andresen-Flores-Morales-2014 , Arbogast-Gros-2019 , Arijs-Rutgeerts-2009 , Arroyo-Aloy-2014 , Arroyo-Aloy-2015 , Asadi-Dhanvantari-2018

**B**

---

Bailey-Hieter-2015 , Bandyopadhyay-Ideker-2010 , Banks-Washburn-2016 , Bantscheff-Drewes-2011 , Barr-Knapp-2009 , Barreiro-Alonso-Cerdán-2018 , Barrios-Rodiles-Wrana-2005 , Behrends-Harper-2010 , Behzadnia-Lührmann-2007 , Benleulmi-Chaachoua-Jockers-2016 A , Benleulmi-Chaachoua-Jockers-2016 B , Bennett-Harper-2010 , Benzinger-Hermeking-2005 , Berggård-James-2006 , Bett-Hay-2013 , Beyer-Boldt-2018 , Bhatnagar-Attie-2014 , Bild-Nevins-2006 B , BIOGRID-SMALL-SCALE-STUDIES , BIOGRID-SMALL-SCALE-STUDIES , Bishof-Seyfried-2018 , Blandin-Richard-2013 , Blomen-Brummelkamp-2015 , Blomen-Brummelkamp-2015 , Bogachek-Weigel-2014 , Boldrick-Relman-2002 , Boldt-Roepman-2016 , Botham-Schimmer-2019 , Bouwmeester-Superti-Furga-2004 , Brady-Omary-2018 , Brajenovic-Drewes-2004 , Brehme-Superti-Furga-2009 , Burington-Shaughnessy-2008 , Butland-Hayden-2014 , Byron-Humphries-2012

**C**

---

Cai-Conaway-2007 , Camargo-Brandon-2007 , Campos-Reinberg-2015 , Cao-Chinnaiyan-2014 , Carmon-Liu-2014 , Caron-van Attikum-2019 , CELL_MAP , Chen-Brown-2002 , Chen-Ge-2013 A , Chen-Ge-2013 B , Chen-Guan-2018 , Chen-Huang-2014 , Chen-Krogan-2018 , Chen-Yu-2018 , Chen-Zhang-2013 , Chen-Zhou-2019 , Cheng-DeCaprio-2017 , Chi-Reed-2018 , Chitale-Richly-2017 , Choi-Beutler-2019 , Choi-Busino-2018 , Choudhury-Michlewski-2017 , Christianson-Kopito-2011 ,

## C

Cloutier-Coulombe-2013 , Cloutier-Coulombe-2017 , Colicelli-2010 , Colland-Gauthier-2004 , Conte-Perez-Oliva-2018 , Cooper-Green-2015 , Corominas-Iakoucheva-2014 , Couzens-Gingras-2013 , Cox-Rizzino-2013 , Coyaud-Raught-2015 , Crow-Cristea-2017

## D

Daakour-Twizere-2016 , Dabbaghizadeh-Tanguay-2018 , Dart-Wells-2015 , Das-Broemer-2019 , Davis-Glaunsinger-2015 , de Hoog-Mann-2004 , Devarajan-Ketha-Kumar-2012 , Diner-Cristea-2015 , Dittmer-Misteli-2014 , Dobbin-Giordano-2005 , Douanne-Bidère-2019 , Drissi-Boisvert-2015 , Du-Krogan-2017

## E

Elliott-Gyrd-Hansen-2016 , Emdal-Olsen-2015 , Enzo-Dupont-2015 , Ertych-Bastians-2016 , Ewing-Figeys-2007

## F

Fang-Lin-2011 , Faust-Frankel-2018 , Fenner-Prehn-2010 , Floyd-Pagliarini-2016 , Foerster-Ritter-2013 , Fogeron-Lange-2013 , Fonseca-Damgaard-2015 , Foster-Marshall-2013 , Fragoza-Yu-2019 , Freibaum-Taylor-2010

## G

Gabriel-Baumgrass-2016 , Gallardo-Vara-Bernabeu-2019 , Galligan-Howley-2015 , Gao-Reinberg-2012 , Gao-Vaziri-2016 , Garzia-Sonenberg-2017 , Gautier-Hall-2009 , Giannone-Liu-2010 , Gilmore-Washburn-2016 , Giurato-Tarallo-2018 , Glatter-Gstaiger-2009 , Gloeckner-Ueffing-2007 , Goehler-Wanker-2004 , Gordon-Krogan-2020 , Goudreault-Gingras-2009 , Greco-Cristea-2011 , Grossmann-Stelzl-2015 , Guarani-Harper-2014 , Guard-Old-2019 , Guardia-Laguarta-Przedborski-2019 , Guderian-Grimmler-2011 , Gupta-Pelletier-2015

## H

Han-Bassik-2017 A , Han-Bassik-2017 B , Hanson-Clayton-2014 , Hauri-Beisel-2016 , Hauri-Gstaiger-2013 , Havrylov-Redowicz-2009 , Havugimana-Emili-2012 , Hayes-Urbé-2012 , Hegele-Stelzl-2012 A , Hegele-Stelzl-2012 B , Heidelberger-Beli-2018 , Hein-Mann-2015 , Hermjakob-Apweiler-2004 , Herr-Helleday-2015 , Hoffmeister-Längst-2017 , Horlbeck-Gilbert-2018 A , Horlbeck-Gilbert-2018 B , Hosp-Selbach-2015 , Hou-Chen-2018 , Hou-Huang-2017 , Hu-Woods-2019 , Hu-Yin-2019 , Hubel-Pichlmair-2019 , Huber-Hoelz-2017 , HUMANCYC , Humphries-Humphries-2009 , Hussain-Aldaz-2018 , Hutchins-Peters-2010 , Huttlin-Gygi-2015 , Huttlin-Harper-2017 , Hüttenhain-Krogan-2019

## I

I2D-BIND-Fly2Human , I2D-BIND-Mouse2Human , I2D-BIND-Rat2Human , I2D-BIND-Worm2Human , I2D-BIND-Yeast2Human , I2D-BioGRID-Fly2Human , I2D-BioGRID-Mouse2Human , I2D-BioGRID-Rat2Human , I2D-BioGRID-Worm2Human , I2D-BioGRID-Yeast2Human , I2D-Chen-Pawson-2009-PiwiScreen-

## I

Mouse2Human , I2D-Formstecher-Daviet-2005-Embryo-Fly2Human , I2D-Formstecher-Daviet-2005-Head-Fly2Human , I2D-Giot-Rothbert-2003-High-Fly2Human , I2D-Giot-Rothbert-2003-Low-Fly2Human , I2D-INNATEDB-Mouse2Human , I2D-IntAct-Fly2Human , I2D-IntAct-Mouse2Human , I2D-IntAct-Rat2Human , I2D-IntAct-Worm2Human , I2D-IntAct-Yeast2Human , I2D-Krogan-Greenblatt-2006-Core-Yeast2Human , I2D-Krogan-Greenblatt-2006-NonCore-Yeast2Human , I2D-Li-Vidal-2004-CE-DATA-Worm2Human , I2D-Li-Vidal-2004-CORE-1-Worm2Human , I2D-Li-Vidal-2004-CORE-2-Worm2Human , I2D-Li-Vidal-2004-interolog-Worm2Human , I2D-Li-Vidal-2004-literature-Worm2Human , I2D-Li-Vidal-2004-non-core-Worm2Human , I2D-Manual-Mouse2Human , I2D-Manual-Rat2Human , I2D-MGI-Mouse2Human , I2D-MINT-Fly2Human , I2D-MINT-Mouse2Human , I2D-MINT-Rat2Human , I2D-MINT-Worm2Human , I2D-MINT-Yeast2Human , I2D-MIPS-Yeast2Human , I2D-Ptacek-Snyder-2005-Yeast2Human , I2D-Stanyon-Finley-2004-CellCycle-Fly2Human , I2D-Tarassov-PCA-Yeast2Human , I2D-Tewari-Vidal-2004-TGFb-Worm2Human , I2D-vonMering-Bork-2002-High-Yeast2Human , I2D-vonMering-Bork-2002-Low-Yeast2Human , I2D-vonMering-Bork-2002-Medium-Yeast2Human , I2D-Wang-Orkin-2006-EScmplx-Mouse2Human , I2D-Wang-Orkin-2006-EScmplxIP-Mouse2Human , I2D-Wang-Orkin-2006-EScmplxlow-Mouse2Human , I2D-Yu-Vidal-2008-GoldStd-Yeast2Human , IMID , Ingham-Pawson-2005 , Innocenti-Brown-2011 , INTERPRO , Iradi-Borchelt-2018 , IREF-bhf-ucl , IREF-bind , IREF-bind-translation , IREF-biogrid , IREF-corum , IREF-dip , IREF-hpidb , IREF-hprd , IREF-huri , IREF-innatedb , IREF-intact , IREF-intcomplex , IREF-matrixdb , IREF-mbinfo , IREF-mint , IREF-mppi , IREF-quickgo , IREF-reactome , IREF-SMALL-SCALE-STUDIES , IREF-SMALL-SCALE-STUDIES , IREF-spike , IREF-uniprotpp , IREF-virushost , Ivanochko-Arrowsmith-2019

## J

Jain-Parker-2016 , Jang-Trono-2018 , Jeronimo-Coulombe-2007 , Jiang-de Kok-2017 , Jin-Pawson-2004 , Jirawatnotai-Sicinski-2011 , Johnson-Kerner-Wichterle-2015 , Johnson-Shoemaker-2003 , Jones-MacBeath-2006 , Joshi-Cristea-2013 , Jozwik-Carroll-2016 , Jäger-Krogan-2011

## K

Kahle-Zoghbi-2011 , Kaltenbach-Hughes-2007 , Kang-Shin-2015 , Karras-Soengas-2019 , Kato-Sternberg-2014 , Katsogiannou-Rocchi-2014 , Kawahara-Paes Leme-2017 , Keller-Lee-2014 , Kennedy-Kolch-2020 A , Kennedy-Kolch-2020 B , Khanna-Parnaik-2018 , Kim-Major-2015 , Kneissl-Grummt-2003 , Koch-Hermeking-2007 , Kotlyar-Jurisica-2015 , Kristensen-Foster-2012 , Kumar-Maddika-2017 , Kumar-Vertegaal-2017 , Kupka-Walczak-2016 , Kärblane-Sarmiento-2015 , Kırlı-Görlich-2015

## L

Lambert-Gingras-2015 , Lampert-Peter-2018 , Lau-Ronai-2012 , Lee-Choi-2016 ,

**L**

---

Lee-Choi-2017 , Lee-Jeong-2017 , Lee-Jou-2019 , Lee-Mayr-2019 , Lee-Songyang-2011 , Lehner-Sanderson-2004 A , Lehner-Sanderson-2004 B , Leung-Jones-2014 , Leung-Miller-2017 , Li-Chen-2015 , Li-Dorf-2011 A , Li-Dorf-2011 B , Li-Dorf-2014 , Li-Fu-2017 , Li-Haura-2013 , Li-Hung-2019 , Li-Lu-2018 , Li-Wang-2016 , Li-Zhou-2017 , Liebelt-Vertegaal-2020 , Lim-Zoghbi-2006 , Lin-Smith-2010 , Lipp-Guthrie-2015 , Liu-Chen-2019 , Liu-Sun-2019 , Liu-Takahashi-2017 , Liu-Tan-2018 , Liu-Varjosalo-2018 , Liu-Wang-2012 , Liu-Xu-2018 , Liu-Yang-2019 , Llères-Lamond-2010 , Loch-Strickler-2012 , Low-Heck-2014 , Lu-Bohr-2017 , Lu-Zhang-2013 , Luck-Calderwood-2020 , Lum-Cristea-2018 , Luo-Elledge-2009

**M**

---

Mak-Moffat-2010 , Malinová-Verheggen-2017 , Mallon-McKay-2013 , Malovannaya-Qin-2010 , Malty-Babu-2017 , Markson-Sanderson-2009 , Martin-Elledge-2017 , Maréchal-Zou-2014 , Matsumoto-Nakayama-2005 , Matsuoka-Elledge-2007 , McCracken-Blencowe-2005 , McFarland-Nussbaum-2008 , McNamara-D'Orso-2016 , Meek-Piwnica-Worms-2004 , Menon-Litovchick-2019 , Milev-Mouland-2012 , Miyamoto-Sato-Yanagawa-2010 , Mohammed-Carroll-2013 , Moon-Kim-2014 , Moutaoufik-Babu-2019 , Mugabo-Lim-2018 , Muller-Demeret-2012 , Murakawa-Landthaler-2015

**N**

---

Nakamura-Groth-2019 , Nakayama-Ohara-2002 , Napolitano-Meroni-2011 , Narayan-Bennett-2012 , Nassa-Weisz-2019 , Nathan-Goldberg-2013 , NCI_NATURE , Neganova-Lako-2011 , Newman-Keating-2003 , Noguchi-Kawahara-2018 , Nowak-Sommer-2019

**O**

---

Oliviero-Cagney-2015 , Oliviero-Cagney-2016 , Olma-Pintard-2009 , Oláh-Ovádi-2011 , Ouyang-Gill-2009

**P**

---

Panigrahi-Pati-2012 , Pankow-Yates-2015 , Pao-Virdee-2018 , Papp-Lamia-2015 , Pech-Settleman-2019 , Perez-Hernandez-Yáñez-Mó-2013 , Perez-Perri-Espinosa-2016 , Perou-Botstein-1999 , Perou-Botstein-2000 , Persaud-Rotin-2009 A , Persaud-Rotin-2009 B , Petschnigg-Stagljar-2014 , PFAM , Phillips-Corn-2013 , Pichlmair-Superti-Furga-2011 , Pichlmair-Superti-Furga-2012 , Pilling-Cooper-2017 , Pladevall-Morera-Lopez-Contreras-2019 , Ptushkina-Ray-2017

**R**

---

Raisner-Gascoigne-2018 , Ramachandran-LaBaer-2004 , Raman-Harper-2015 , Ramaswamy-Golub-2001 , Ravasi-Hayashizaki-2010 , REACTOME , Reinke-Keating-2010 , Reinke-Keating-2013 , Rengasamy-Walsh-2017 , Reyniers-Taymans-2014 , Richter-Chrzanowska-Lightowlers-2010 , Rieger-Chu-2004 , Rivera-Paes Leme-2018 , Rodriguez-von Kriegsheim-2016 , Roewenstrunk-de la Luna-2019 , Rolland-Vidal-2014 , Rosenbluh-Hahn-2016 , Rosenwald-Staudt-2001 , Ross-Perou-

**R**

---

2001 , Roth-Zlotnik-2006 , Rowbotham-Mermoud-2011 , Roy-Pardo-2014 , Roy-Parent-2013 , Rual-Vidal-2005

**S**

---

Saez-Vilchez-2018 , Sahni-Vidal-2015 , Saito-Kobarg-2017 , Sala-Ampe-2017 , Salvetti-Greco-2016 , Sang-Jackson-2011 , Sato-Conaway-2004 , Savidis-Brass-2016 , Schadt-Shoemaker-2004 , Schiza-Diamandis-2018 , Scholz-Taylor-2016 , Scifo-Lalowski-2015 , Scott-Guy-2017 , Scott-Schulman-2016 , Shami Shah-Baskin-2019 , Shen-Chen-2019 , Shen-Mali-2017 , Sherman-Teitell-2010 , Simabuco-Zanchin-2019 , Singh-Moore-2012 , So-Colwill-2015 , Sokolina-Stagljar-2017 , Soler-López-Aloy-2011 , Sowa-Harper-2009 , Srivas-Ideker-2016 , St-Denis-Gingras-2015 , St-Denis-Gingras-2016 , Stehling-Lill-2012 , Stehling-Lill-2013 , Stelzl-Wanker-2005 , Stuart-Kim-2003 , Sundell-Ivarsson-2018 , Suter-Wanker-2013 , Swayampakula-Dedhar-2017

**T**

---

Taipale-Lindquist-2012 , Taipale-Lindquist-2014 , Takahashi-Conaway-2011 , Tang-Wang-2019 , Tarallo-Weisz-2011 , Teixeira-Gomes-2010 , Teixeira-Laman-2016 A , Teixeira-Laman-2016 B , Thalappilly-Dusetti-2008 , Thompson-Luchansky-2014 , Tiemann-Kani-2019 , Tomkins-Manzoni-2018 , Tong-Moran-2014 , Toyoshima-Grandori-2012 , Trepte-Wanker-2018 A , Trepte-Wanker-2018 B , Tsai-Cristea-2012

**U**

---

Ugidos-Vandenbroeck-2019

**V**

---

Van Acker-Dewilde-2019 , Van Alstyne-Pellizzoni-2018 , Van Quickelberghe-Gevaert-2018 , van Wijk-Timmers-2009 , Vandamme-Angrand-2011 , Varier-Vermeulen-2016 , Varjosalo-Gstaiger-2013 A , Varjosalo-Gstaiger-2013 B , Varjosalo-Superti-Furga-2013 , Vastrik-Stein-2007 , Venkatesan-Vidal-2009 , Viita-Vartiainen-2019 , Vinayagam-Wanker-2011 , Virok-Fülöp-2011 , Vizeacoumar-Moffat-2013 , von Hundelshausen-Weber-2017

**W**

---

Wallach-Kramer-2013 , Wan-Emili-2015 , Wang-Balch-2006 , Wang-Cheung-2015 , Wang-He-2008 , Wang-Huang-2017 , Wang-Liu-2019 , Wang-Maris-2006 , Wang-Xiong-2019 , Wang-Xu-2015 , Wang-Yang-2011 , Watanabe-Fujita-2018 , Weimann-Stelzl-2013 A , Weimann-Stelzl-2013 B , Weinmann-Meister-2009 , Weishäupl-Schmidt-2019 , Weith-Meyer-2018 , Whisenant-Salomon-2015 , Wilkinson-Coba-2019 , Willingham-Muchowski-2003 , Winczura-Jensen-2018 , Wong-O'Bryan-2012 , Woods-Monteiro-2012 A , Woods-Monteiro-2012 B , Woodsmith-Sanderson-2012 , Wu-Garvey-2007 , Wu-Li-2007 , Wu-Ma-2012 , Wu-Stein-2010 , Wu-Stein-2010

**X**

---

Xiao-Brown-2018 , Xiao-Lefkowitz-2007 , Xie-Cong-2013 , Xie-Green-2012 , Xie-

**X**

Zhang-2017 , Xu-Ye-2012 , Xu-Zetter-2016

**Y**

Yachie-Roth-2016 , Yadav-Varjosalo-2017 , Yamauchi-Maeda-2018 , Yang-Brasier-2015 , Yang-Chen-2010 , Yang-Maurer-2018 , Yang-Vidal-2016 , Yang-Wang-2018 , Yao-Stagljar-2017 A , Yao-Stagljar-2017 B , Yatim-Benkirane-2012 , Yeung-Dougan-2019 , Yu-Chow-2013 , Yu-Engel-2018 , Yu-Vidal-2011 , Yue-Liu-2018

**Z**

Zanon-Pichler-2013 , Zeller-Wei-2006 , Zhang-Shang-2006 , Zhang-Vermeulen-2017 , Zhang-Wang-2018 , Zhang-Wheeler-2014 , Zhang-Xu-2018 , Zhang-Zou-2011 , Zhao-Krug-2005 , Zhao-Yang-2011 , Zhong-Vidal-2016 , Zhou-Conrads-2004 , Zhou-Hanemann-2016 , Zhu-Liu-2018

# Genes

| Gene | Description | Rank |
|---|---|---|
| RNASE13 | ribonuclease A family member 13 (inactive) [Source:HGNC Symbol;Acc:HGNC:25285] | N/A |
| TNFRSF13C | TNF receptor superfamily member 13C [Source:HGNC Symbol;Acc:HGNC:17755] | N/A |
| CRHR1 | corticotropin releasing hormone receptor 1 [Source:HGNC Symbol;Acc:HGNC:2357] | N/A |
| TIRAP | TIR domain containing adaptor protein [Source:HGNC Symbol;Acc:HGNC:17192] | N/A |
| SLC39A10 | solute carrier family 39 member 10 [Source:HGNC Symbol;Acc:HGNC:20861] | N/A |
| TNFSF13B | TNF superfamily member 13b [Source:HGNC Symbol;Acc:HGNC:11929] | N/A |
| WNT3A | Wnt family member 3A [Source:HGNC Symbol;Acc:HGNC:15983] | N/A |
| MTHFR | methylenetetrahydrofolate reductase [Source:HGNC Symbol;Acc:HGNC:7436] | N/A |
| TNFRSF4 | TNF receptor superfamily member 4 [Source:HGNC Symbol;Acc:HGNC:11918] | N/A |
| ATAD5 | ATPase family AAA domain containing 5 [Source:HGNC Symbol;Acc:HGNC:25752] | N/A |
| IL21 | interleukin 21 [Source:HGNC Symbol;Acc:HGNC:6005] | N/A |
| CLCF1 | cardiotrophin like cytokine factor 1 [Source:HGNC Symbol;Acc:HGNC:17412] | N/A |
| BMI1 | BMI1 proto-oncogene, polycomb ring finger [Source:HGNC Symbol;Acc:HGNC:1066] | N/A |
| LMO3 | LIM domain only 3 [Source:HGNC Symbol;Acc:HGNC:6643] | N/A |
| OXTR | oxytocin receptor [Source:HGNC Symbol;Acc:HGNC:8529] | N/A |
| CD320 | CD320 molecule [Source:HGNC Symbol;Acc:HGNC:16692] | N/A |
| OPRM1 | opioid receptor mu 1 [Source:HGNC Symbol;Acc:HGNC:8156] | N/A |
| NFATC2 | nuclear factor of activated T cells 2 [Source:HGNC Symbol;Acc:HGNC:7776] | N/A |
| IL4 | interleukin 4 [Source:HGNC Symbol;Acc:HGNC:6014] | N/A |
| DRD3 | dopamine receptor D3 [Source:HGNC Symbol;Acc:HGNC:3024] | N/A |
| CARD11 | caspase recruitment domain family member 11 [Source:HGNC Symbol;Acc:HGNC:16393] | N/A |

| Gene | Description | Rank |
| --- | --- | --- |
| STXBP5L | syntaxin binding protein 5 like [Source:HGNC Symbol;Acc:HGNC:30757] | N/A |
| COMT | catechol-O-methyltransferase [Source:HGNC Symbol;Acc:HGNC:2228] | N/A |
| BST1 | bone marrow stromal cell antigen 1 [Source:HGNC Symbol;Acc:HGNC:1118] | N/A |
| IL13 | interleukin 13 [Source:HGNC Symbol;Acc:HGNC:5973] | N/A |
| EPHB2 | EPH receptor B2 [Source:HGNC Symbol;Acc:HGNC:3393] | N/A |
| IL10 | interleukin 10 [Source:HGNC Symbol;Acc:HGNC:5962] | N/A |
| SLC6A3 | solute carrier family 6 member 3 [Source:HGNC Symbol;Acc:HGNC:11049] | N/A |
| IL3 | interleukin 3 [Source:HGNC Symbol;Acc:HGNC:6011] | N/A |
| SLC6A4 | solute carrier family 6 member 4 [Source:HGNC Symbol;Acc:HGNC:11050] | N/A |
| TICAM1 | toll like receptor adaptor molecule 1 [Source:HGNC Symbol;Acc:HGNC:18348] | N/A |
| VAV3 | vav guanine nucleotide exchange factor 3 [Source:HGNC Symbol;Acc:HGNC:12659] | N/A |
| CD40LG | CD40 ligand [Source:HGNC Symbol;Acc:HGNC:11935] | N/A |
| CHRNB2 | cholinergic receptor nicotinic beta 2 subunit [Source:HGNC Symbol;Acc:HGNC:1962] | N/A |
| IL6R | interleukin 6 receptor [Source:HGNC Symbol;Acc:HGNC:6019] | N/A |
| CACNA1C | calcium voltage-gated channel subunit alpha1 C [Source:HGNC Symbol;Acc:HGNC:1390] | N/A |
| PRLR | prolactin receptor [Source:HGNC Symbol;Acc:HGNC:9446] | N/A |
| FKBP5 | FKBP prolyl isomerase 5 [Source:HGNC Symbol;Acc:HGNC:3721] | N/A |
| DRD4 | dopamine receptor D4 [Source:HGNC Symbol;Acc:HGNC:3025] | N/A |
| IL5 | interleukin 5 [Source:HGNC Symbol;Acc:HGNC:6016] | N/A |
| CD38 | CD38 molecule [Source:HGNC Symbol;Acc:HGNC:1667] | N/A |
| NCKAP1L | NCK associated protein 1 like [Source:HGNC Symbol;Acc:HGNC:4862] | N/A |
| DRD2 | dopamine receptor D2 [Source:HGNC Symbol;Acc:HGNC:3023] | N/A |
| IRS2 | insulin receptor substrate 2 [Source:HGNC Symbol;Acc:HGNC:6126] | N/A |
| TLR4 | toll like receptor 4 [Source:HGNC Symbol;Acc:HGNC:11850] | N/A |
| PELI1 | pellino E3 ubiquitin protein ligase 1 [Source:HGNC Symbol;Acc:HGNC:8827] | N/A |
| CRHBP | corticotropin releasing hormone binding protein [Source:HGNC Symbol; | N/A |

| Gene | Description | Rank |
|---|---|---|
| | Acc:HGNC:2356] | |
| IL2 | interleukin 2 [Source:HGNC Symbol;Acc:HGNC:6001] | N/A |
| SASH3 | SAM and SH3 domain containing 3 [Source:HGNC Symbol;Acc:HGNC:15975] | N/A |
| GPR183 | G protein-coupled receptor 183 [Source:HGNC Symbol;Acc:HGNC:3128] | N/A |
| MAOA | monoamine oxidase A [Source:HGNC Symbol;Acc:HGNC:6833] | N/A |
| BDNF | brain derived neurotrophic factor [Source:HGNC Symbol;Acc:HGNC:1033] | N/A |
| TLR9 | toll like receptor 9 [Source:HGNC Symbol;Acc:HGNC:15633] | N/A |
| NPY | neuropeptide Y [Source:HGNC Symbol;Acc:HGNC:7955] | N/A |
| ADA | adenosine deaminase [Source:HGNC Symbol;Acc:HGNC:186] | N/A |
| CD40 | CD40 molecule [Source:HGNC Symbol;Acc:HGNC:11919] | N/A |
| IL6ST | interleukin 6 signal transducer [Source:HGNC Symbol;Acc:HGNC:6021] | N/A |
| TCF3 | transcription factor 3 [Source:HGNC Symbol;Acc:HGNC:11633] | N/A |
| MIF | macrophage migration inhibitory factor [Source:HGNC Symbol;Acc:HGNC:7097] | N/A |
| IL7 | interleukin 7 [Source:HGNC Symbol;Acc:HGNC:6023] | N/A |
| TFRC | transferrin receptor [Source:HGNC Symbol;Acc:HGNC:11763] | N/A |
| BCL6 | BCL6 transcription repressor [Source:HGNC Symbol;Acc:HGNC:1001] | N/A |
| CD74 | CD74 molecule [Source:HGNC Symbol;Acc:HGNC:1697] | N/A |
| BCL2 | BCL2 apoptosis regulator [Source:HGNC Symbol;Acc:HGNC:990] | N/A |
| MEF2C | myocyte enhancer factor 2C [Source:HGNC Symbol;Acc:HGNC:6996] | N/A |
| IL6 | interleukin 6 [Source:HGNC Symbol;Acc:HGNC:6018] | N/A |
| TNFRSF1B | TNF receptor superfamily member 1B [Source:HGNC Symbol;Acc:HGNC:11917] | N/A |
| CD81 | CD81 molecule [Source:HGNC Symbol;Acc:HGNC:1701] | N/A |
| APOE | apolipoprotein E [Source:HGNC Symbol;Acc:HGNC:613] | N/A |
| PTPRC | protein tyrosine phosphatase receptor type C [Source:HGNC Symbol;Acc:HGNC:9666] | N/A |
| TNF | tumor necrosis factor [Source:HGNC Symbol;Acc:HGNC:11892] | N/A |
| TNFRSF1A | TNF receptor superfamily member 1A [Source:HGNC Symbol;Acc:HGNC:11916] | N/A |
| BTK | Bruton tyrosine kinase [Source:HGNC Symbol;Acc:HGNC:1133] | N/A |
| CDKN1A | cyclin dependent kinase inhibitor 1A [Source:HGNC Symbol;Acc:HGNC:1784] | N/A |

| Gene | Description | Rank |
|---|---|---|
| TCN2 | transcobalamin 2 [Source:HGNC Symbol;Acc:HGNC:11653] | 1 |
| IL2RG | interleukin 2 receptor subunit gamma [Source:HGNC Symbol;Acc: HGNC:6010] | 2 |
| IL9 | interleukin 9 [Source:HGNC Symbol;Acc:HGNC:6029] | 3 |
| NCKAP1 | NCK associated protein 1 [Source:HGNC Symbol;Acc:HGNC:7666] | 4 |
| AKAP1 | A-kinase anchoring protein 1 [Source:HGNC Symbol;Acc:HGNC:367] | 5 |
| IL7R | interleukin 7 receptor [Source:HGNC Symbol;Acc:HGNC:6024] | 6 |
| CNTF | ciliary neurotrophic factor [Source:HGNC Symbol;Acc:HGNC:2169] | 7 |
| CHRNA4 | cholinergic receptor nicotinic alpha 4 subunit [Source:HGNC Symbol; Acc:HGNC:1958] | 8 |
| TICAM2 | toll like receptor adaptor molecule 2 [Source:HGNC Symbol;Acc:HGNC: 21354] | 9 |
| IFITM1 | interferon induced transmembrane protein 1 [Source:HGNC Symbol; Acc:HGNC:5412] | 10 |
| IL10RA | interleukin 10 receptor subunit alpha [Source:HGNC Symbol;Acc: HGNC:5964] | 11 |
| PELI3 | pellino E3 ubiquitin protein ligase family member 3 [Source:HGNC Symbol;Acc:HGNC:30010] | 12 |
| HGF | hepatocyte growth factor [Source:HGNC Symbol;Acc:HGNC:4893] | 13 |
| PELI2 | pellino E3 ubiquitin protein ligase family member 2 [Source:HGNC Symbol;Acc:HGNC:8828] | 14 |
| WLS | Wnt ligand secretion mediator [Source:HGNC Symbol;Acc:HGNC: 30238] | 15 |
| DPP4 | dipeptidyl peptidase 4 [Source:HGNC Symbol;Acc:HGNC:3009] | 16 |
| SIGIRR | single Ig and TIR domain containing [Source:HGNC Symbol;Acc: HGNC:30575] | 17 |
| IL2RA | interleukin 2 receptor subunit alpha [Source:HGNC Symbol;Acc:HGNC: 6008] | 18 |
| TLR6 | toll like receptor 6 [Source:HGNC Symbol;Acc:HGNC:16711] | 19 |
| NTF4 | neurotrophin 4 [Source:HGNC Symbol;Acc:HGNC:8024] | 20 |

# Networks

## Co-expression 47.49%

### Bild-Nevins-2006 B 6.58%

Oncogenic pathway signatures in human cancers as a guide to targeted therapies. Bild et al (2006). *Nature*

Co-expression with 285,368 interactions from GEO

### Boldrick-Relman-2002 5.37%

Stereotyped and specific gene expression programs in human innate immune responses to bacteria. Boldrick et al (2002). *Proc Natl Acad Sci U S A*

Co-expression with 116,197 interactions from supplementary material

### Dobbin-Giordano-2005 4.83%

Interlaboratory comparability study of cancer gene expression analysis using oligonucleotide microarrays. Dobbin et al (2005). *Clin Cancer Res*

Co-expression with 452,322 interactions from GEO

### Mallon-McKay-2013 3.93%

StemCellDB: the human pluripotent stem cell database at the National Institutes of Health. Mallon et al (2013). *Stem Cell Res*

Co-expression with 602,113 interactions from GEO

### Arijs-Rutgeerts-2009 3.71%

Mucosal gene expression of antimicrobial peptides in inflammatory bowel disease before and after first infliximab treatment. Arijs et al (2009). *PLoS One*

Co-expression with 676,695 interactions from GEO

### Roth-Zlotnik-2006 3.23%

Gene expression analyses reveal molecular relationships among 20 regions of the human CNS. Roth et al (2006). *Neurogenetics*

Co-expression with 683,844 interactions from GEO

### Wang-Maris-2006 2.88%

Integrative genomics identifies distinct molecular classes of neuroblastoma and shows that multiple genes are targeted by regional alterations in DNA copy number. Wang et al (2006). *Cancer Res*

Co-expression with 270,388 interactions from GEO

### Burington-Shaughnessy-2008 2.35%

Tumor cell gene expression changes following short-term in vivo exposure to single agent chemotherapeutics are related to survival in multiple myeloma. Burington et al (2008). *Clin Cancer Res*

Co-expression with 295,320 interactions from GEO

### Chen-Brown-2002 1.86%

Gene expression patterns in human liver cancers. Chen et al (2002). *Mol Biol Cell*

Co-expression with 291,300 interactions from supplementary material

### Ramaswamy-Golub-2001 1.74%

Multiclass cancer diagnosis using tumor gene expression signatures. Ramaswamy et al (2001). *Proc Natl Acad Sci U S A*

Co-expression with 284,829 interactions from supplementary material

### Alizadeh-Staudt-2000 1.61%

Distinct types of diffuse large B-cell lymphoma identified by gene expression profiling. Alizadeh et al (2000). *Nature*

Co-expression with 92,360 interactions from supplementary material

## Co-expression 47.49%

### Perou-Botstein-2000 1.58%

Molecular portraits of human breast tumours. Perou et al (2000). *Nature*

Co-expression with 189,373 interactions from supplementary material

### Ross-Perou-2001 1.49%

A comparison of gene expression signatures from breast tumors and breast tissue derived cell lines. Ross et al (2001). *Dis Markers*

Co-expression with 146,858 interactions from supplementary material

### Wang-Cheung-2015 1.17%

Genetic variation in insulin-induced kinase signaling. Wang et al (2015). *Mol Syst Biol*

Co-expression with 422,896 interactions from GEO

### Rieger-Chu-2004 1.13%

Toxicity from radiation therapy associated with abnormal transcriptional responses to DNA damage. Rieger et al (2004). *Proc Natl Acad Sci U S A*

Co-expression with 266,879 interactions from GEO

### Rosenwald-Staudt-2001 1.13%

Relation of gene expression phenotype to immunoglobulin mutation genotype in B cell chronic lymphocytic leukemia. Rosenwald et al (2001). *J Exp Med*

Co-expression with 118,097 interactions from supplementary material

### Innocenti-Brown-2011 1.04%

Identification, replication, and functional fine-mapping of expression quantitative trait loci in primary human liver tissue. Innocenti et al (2011). *PLoS Genet*

Co-expression with 620,205 interactions from GEO

### Wu-Garvey-2007 0.94%

The effect of insulin on expression of genes and biochemical pathways in human skeletal muscle. Wu et al (2007). *Endocrine*

Co-expression with 275,155 interactions from GEO

### Jiang-de Kok-2017 0.91%

Omics-based identification of the combined effects of idiosyncratic drugs and inflammatory cytokines on the development of drug-induced liver injury. Jiang et al (2017). *Toxicol Appl Pharmacol*

Co-expression with 444,959 interactions from GEO

## Physical Interactions 19.12%

### IREF-reactome 3.88%

Physical Interactions with 111,926 interactions from iRefIndex

### Vastrik-Stein-2007 3.88%

Reactome: a knowledge base of biologic pathways and processes. Vastrik et al (2007). *Genome Biol*

Physical Interactions with 111,926 interactions from iRefIndex

### IREF-bind 2.07%

Physical Interactions with 3,524 interactions from iRefIndex

### Moutaoufik-Babu-2019 1.72%

Rewiring of the Human Mitochondrial Interactome during Neuronal Reprogramming Reveals Regulators of the Respirasome and Neurogenesis. Moutaoufik et al (2019). *iScience*

Physical Interactions with 6,357 interactions from BioGRID

## Physical Interactions 19.12%

### IREF-quickgo 1.65%

Physical Interactions with 9,985 interactions from iRefIndex

### Bouwmeester-Superti-Furga-2004 1.28%

A physical and functional map of the human TNF-alpha/NF-kappa B signal transduction pathway. Bouwmeester et al (2004). *Nat Cell Biol*

Physical Interactions with 1,694 interactions from iRefIndex

### IREF-innatedb 0.96%

Physical Interactions with 2,355 interactions from iRefIndex

### IREF-uniprotpp 0.94%

Physical Interactions with 2,470 interactions from iRefIndex

### IREF-bind-translation 0.92%

Physical Interactions with 6,056 interactions from iRefIndex

### Bhatnagar-Attie-2014 0.82%

Phosphorylation and degradation of tomosyn-2 de-represses insulin secretion. Bhatnagar et al (2014). *J Biol Chem*

Physical Interactions with 198 interactions from BioGRID

### Kupka-Walczak-2016 0.55%

SPATA2-Mediated Binding of CYLD to HOIP Enables CYLD Recruitment to Signaling Complexes. Kupka et al (2016). *Cell Rep*

Physical Interactions with 411 interactions from BioGRID

### IREF-mint 0.32%

Physical Interactions with 14,408 interactions from iRefIndex

### Huttlin-Gygi-2015 0.08%

The BioPlex Network: A Systematic Exploration of the Human Interactome. Huttlin et al (2015). *Cell*

Physical Interactions with 23,384 interactions from BioGRID

### BIOGRID-SMALL-SCALE-STUDIES 0.05%

Physical Interactions with 79,201 interactions from BioGRID

## Predicted 13.82%

### I2D-MINT-Rat2Human 7.53%

MINT: a Molecular INTeraction database. Zanzoni et al (2002). *FEBS Lett*

Predicted with 540 interactions from I2D

### I2D-INNATEDB-Mouse2Human 3.69%

InnateDB: facilitating systems-level analyses of the mammalian innate immune response. Lynn et al (2008). *Mol Syst Biol*

Predicted with 4,049 interactions from I2D

### I2D-IntAct-Rat2Human 1.30%

The IntAct molecular interaction database in 2010. Aranda et al (2010). *Nucleic Acids Res*

Predicted with 1,690 interactions from I2D

### I2D-IntAct-Mouse2Human 1.11%

The IntAct molecular interaction database in 2010. Aranda et al (2010). *Nucleic Acids Res*

Predicted with 11,478 interactions from I2D

## Predicted 13.82%

### I2D-BioGRID-Mouse2Human 0.19%

BioGRID: a general repository for interaction datasets. Stark et al (2006). *Nucleic Acids Res*

Predicted with 10,524 interactions from I2D

## Co-localization 8.01%

### Chen-Huang-2014 2.86%

Using an in situ proximity ligation assay to systematically profile endogenous protein-protein interactions in a pathway network. Chen et al (2014). *J Proteome Res*

Co-localization with 559 interactions from BioGRID

### Schadt-Shoemaker-2004 2.58%

A comprehensive transcript index of the human genome generated using microarrays and computational approaches. Schadt et al (2004). *Genome Biol*

Co-localization with 59,920 interactions from GEO

### Johnson-Shoemaker-2003 2.57%

Genome-wide survey of human alternative pre-mRNA splicing with exon junction microarrays. Johnson et al (2003). *Science*

Co-localization with 426,464 interactions from GEO

## Shared protein domains 7.79%

### INTERPRO 7.79%

Shared protein domains with 621,159 interactions from InterPro

## Pathway 3.18%

### Wu-Stein-2010 2.47%

A human functional protein interaction network and its application to cancer data analysis. Wu et al (2010). *Genome Biol*

Pathway with 78,117 interactions from supplementary material

### NCI_NATURE 0.71%

Pathway with 10,118 interactions from Pathway Commons

## Genetic Interactions 0.60%

### Lin-Smith-2010 0.42%

A genome-wide map of human genetic interactions inferred from radiation hybrid genotypes. Lin et al (2010). *Genome Res*

Genetic Interactions with 4,805,334 interactions from supplementary material

### BIOGRID-SMALL-SCALE-STUDIES 0.17%

Genetic Interactions with 651 interactions from BioGRID
